# Comparative 3D genome architecture in vertebrates

**DOI:** 10.1186/s12915-022-01301-7

**Published:** 2022-05-06

**Authors:** Diyan Li, Mengnan He, Qianzi Tang, Shilin Tian, Jiaman Zhang, Yan Li, Danyang Wang, Long Jin, Chunyou Ning, Wei Zhu, Silu Hu, Keren Long, Jideng Ma, Jing Liu, Zhihua Zhang, Mingzhou Li

**Affiliations:** 1 Institute of Animal Genetics and Breeding, College of Animal Science and Technology, Sichuan Agricultural University, Chengdu, 611130 China; 2 Department of Ecology, Tibetan Centre for Ecology and Conservation at WHU-TU, Hubei Key Laboratory of Cell Homeostasis, College of Life Sciences, Wuhan University, Wuhan, 430072 China; 3 Novogene Bioinformatics Institute, Beijing, 100000 China; 4 CAS Key Laboratory of Genome Sciences and Information, Beijing Institute of Genomics, Chinese Academy of Sciences and China National Center for Bioinformation, Beijing, 100101 China; 5 University of Chinese Academy of Sciences, Beijing, 100049 China; 6 School of Artificial Intelligence, University of Chinese Academy of Sciences, Beijing, 100049 China

**Keywords:** Chromatin architecture, Gene expression, Evolution, Vertebrates

## Abstract

**Background:**

The three-dimensional (3D) architecture of the genome has a highly ordered and hierarchical nature, which influences the regulation of essential nuclear processes at the basis of gene expression, such as gene transcription. While the hierarchical organization of heterochromatin and euchromatin can underlie differences in gene expression that determine evolutionary differences among species, the way 3D genome architecture is affected by evolutionary forces within major lineages remains unclear. Here, we report a comprehensive comparison of 3D genomes, using high resolution Hi-C data in fibroblast cells of fish, chickens, and 10 mammalian species.

**Results:**

This analysis shows a correlation between genome size and chromosome length that affects chromosome territory (CT) organization in the upper hierarchy of genome architecture, whereas lower hierarchical features, including local transcriptional availability of DNA, are selected through the evolution of vertebrates. Furthermore, conservation of topologically associating domains (TADs) appears strongly associated with the modularity of expression profiles across species. Additionally, LINE and SINE transposable elements likely contribute to heterochromatin and euchromatin organization, respectively, during the evolution of genome architecture.

**Conclusions:**

Our analysis uncovers organizational features that appear to determine the conservation and transcriptional regulation of functional genes across species. These findings can guide ongoing investigations of genome evolution by extending our understanding of the mechanisms shaping genome architecture.

**Supplementary Information:**

The online version contains supplementary material available at 10.1186/s12915-022-01301-7.

## Background

The evolution of gene regulation is considered to be a main driver of both speciation and adaptation [1]. A growing body of in vivo evidence has shown that the eukaryotic genome forms a highly ordered, hierarchical structure in the interphase nucleus that is closely correlated with, and may even be causally linked to transcriptional machinery that enables appropriate gene expression [2]. At the top of this hierarchy, each chromosome occupies discrete regions, termed chromosome territories (CTs), in the nucleus [3]. At the sub-chromosomal level, the condensed heterochromatin and lightly packed euchromatin are separated into two compartments [4] which may be further divided into smaller sub-compartments [5]. Within these compartments, the chromatin is organized into domain structures that may or may not be nested, such as the compartment domain [6], the loop domain [6], and topologically associating domains (TADs) [7], which range in length from several hundred kilobases to megabases. In particular, TADs have drawn considerable attention in recent studies in which they have been delineated as largely invariant features across cell types and species [8, 9]. Finally, distal chromatin loci, which can span hundreds of kilobases, may stably contact each other to form so-called chromatin loops [5]. Chromatin loops have been proposed as essential components of gene regulation by both facilitating and constraining promoter-enhancer interactions [10].

Transposable elements (TEs) have been found to comprise nearly half of some vertebrate genomes and are divided into two classes (Class I, or retrotransposons, which includes SINE, LINE, and LTR family TEs; and Class II, or DNA transposons) based on their mechanism of transposition. TEs (especially active SINEs) have been reported to be involved in the spatial organization of chromatin in human, mouse, and Drosophila genomes, indicating a role in maintenance and/or reshaping of genome architecture [11]. TEs can mediate small-scale changes in linkage groups but can also lead to large, structural genomic variations, and have contributed to numerous changes in transcriptional regulation [12].

Efforts have been made to characterize the dynamics of genome architecture between cell types and during normal development and disease [13–15]. Nonetheless, differences in genome architecture among different species have remained largely undefined and the elucidation of which could be informative towards understanding the evolution of regulatory mechanisms that drive speciation. This long-standing question motivated earlier studies to compare TAD structures across different species which revealed that individual TAD boundaries are largely conserved within phylogenetic lineages [7]. A recent comparison among bird genomes suggested a strong natural selection pressure on vulnerability of TAD boundaries to DNA double-strand breaks [16], which further supported that likelihood that TADs represent ancient features and that are conserved against evolutionary disruption [9, 17–19]. Two methodological reports presenting inter-species comparisons of 3D genome organization have facilitated side-by-side genome-wide visualization of contact maps between different species [20, 21]. However, to date, no such comparisons of 3D genome organization have simultaneously investigated the differences among 12 vertebrate species, including fish, birds, and mammals. In fact, the majority of previous genome architecture studies limited their analyses to evolutionarily close (typically primate) species [21–23], or examined pairs or small numbers of species [5, 7, 18, 20], mainly focusing on one level of hierarchical organization (most frequently TADs).

**Fig. 1:**
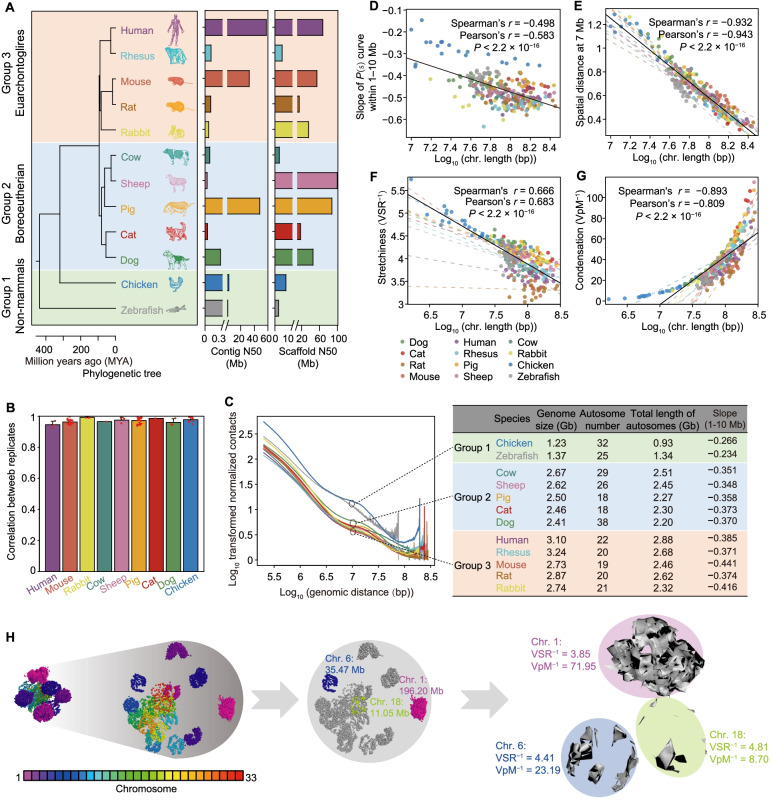
Chromosome lengths may affect overall chromosomal architecture across vertebrates. **A** Summary of the reference genomes of 12 vertebrates. Left panel: The divergence times (million years ago, MYA) and phylogenetic topology of the 12 species. Right panel: N50 values of the genome assemblies were calculated using fragments longer than 500 bp. Species were empirically classified into three groups based on their phylogeny relationship and intra-chromosomal long-range contact frequency decay profile along chromosomal distance. **B** Estimated similarities of intra-chromosomal contact maps between biological replicates for each species at 20 kb resolution using the pairwise stratum-adjusted correlation coefficient (SCC). Data are presented as mean ± SD. The dots on the bars represent the replicates. **C***P*(s) curves (at 100 kb resolution) averaged across all autosomes in the genome of each species. **D–G** Correlations between autosome length and **D** the fitted slope of long-range contact frequency, **E** the spatial distance (*s* = 7-Mb), **F** the stretchiness, and **G** condensation across species. **H** Examples of 3D chromosome conformations in the nucleus of chicken cells

To comprehensively explore the evolutionary principles governing 3D genome architecture and to assess the contributions of genome architecture to transcriptional regulation across species, we performed comparative analyses of high-throughput chromosome conformation capture (Hi-C) in fibroblast cells of 12 vertebrates (Fig. 1A). We selected 12 vertebrate species including human, the 4 important model animals (rhesus macaque, mouse, rat and zebrafish), the 5 agricultural animal species with the highest stock numbers (i.e., pig, chicken, sheep, cow and rabbit), and the 2 major companion animals (i.e., cat and dog). The 10 mammal species cover almost all major evolutionary branches, including primates (i.e., human and rhesus), rodents (i.e., mouse and rat,), lagomorpha (i.e., rabbit), artiodactyla (i.e., pig, cow, and sheep), and carnivora (i.e., cat and dog). Chicken and zebrafish serve as relatively close- and distantly-related outgroups, respectively. The phylogenetic tree of the 12 species (Fig. 1A) was retrieved from the TimeTree database [24]. Our results showed that the genome size and chromosome lengths affect the overall features of 3D genome architecture, e.g., the layout of CTs, while the local features, e.g., the activity or insulation of genome fragments, were evolved with the speciation. These findings illustrate the biophysical dynamics of chromosome distribution in the nucleus and suggested a conservation at the TAD level of the hierarchy of chromosome architecture. The data presented in this work can serve as a starting point for extended research into 3D genome evolution in vertebrates and provide a rich resource for the genomic evolution research community.

## Results

### Initial characteristics of chromosomal conformation across species

In order to explore chromosomal conformation profiles of diverse vertebrates, we performed Hi-C experiments in 25 fibroblasts from 11 vertebrates using 1 to 5 biological replicates (distinct cell lines or primary cells derived from different individuals) for each species [25] (Fig. 1A, Additional file 1: Table S1), which produced a total of ~ 5.75 billion uniquely aligned contacts, with an average depth of ~ 230 million (M) contacts per library (ranging from ~ 102 M for zebrafish, with a relatively small genome size of ~ 1.23 Gb, to ~ 442 M for mouse with a genome size of ~ 2.73 Gb) (Additional file 1: Table S2). We also combined the publicly available Hi-C data of porcine fibroblasts derived from four individuals [26] and two mouse fibroblast cell lines [27] (Additional file 1: Table S1). Among a total of 31 Hi-C libraries, ~ 63.03% were intra-chromosomal contacts, of which ~ 74.40% occurred within 10 Mb (Additional file 1: Table S3, Additional file 2: Fig. S1A). After iterative correction and eigenvector decomposition [28] and quantile normalization [29], we generated 31 Hi-C contact maps at 20-kb resolution (~ 83.12% of bins had at least 1000 intra-chromosomal contacts; Additional file 1: Table S4) [5], which were highly reproducible between biological replicates (median stratum-adjusted correlation coefficient, SCC > 0.905; Fig. 1B).

We plotted curves of the dependence of contact probability (*P*) for the genomic distance (s), *P*(s). Based on these *P*(s) curves, we were able to classify these 12 species into three distinct groups (euarchontoglires, boreoeutherian, and non-mammals) (Fig. 1A). We observed a strong decrease in contact probability with an increase in the distance between loci. The interaction patterns were found different between mammals and non-mammals, i.e., a remarkably high frequency long-range (1–10 Mb) contact was observed in non-mammals, indicated by a steeper decay in *P*(s) curves among mammals (ranging from *s*^−0.348^ for cattle to *s*^−0.441^ for mice) compared to that of chicken (*s*^−0.234^) and zebrafish (*s*^−0.266^) (Fig. 1C).

Notably, we observed a universally negative correlation (Spearman’s *r* = − 0.498, *P* < 2.2 × 10^−16^) between the slops of *P*(s) curves and average length of autosomes across species (Fig. 1D). For example, the long-range contact frequency of the chicken and zebrafish, which have relatively shorter chromosome lengths, is 1.408-fold higher than that of rabbit, human, rat, and mouse (Wilcoxon rank sum test, *P* < 2.2 × 10^–16^; Additional file 2: Fig. S1B). Another example is dog, which had a genome size comparable to other mammals, but a considerably higher chromosome number, and consequently, also had the shortest single chromosomes of the 10 mammals examined here. Thus, we observed the highest long-range contact frequency in the dog genome compared with that in the other mammals (Fig. 1C). These results suggested that the lower long-range contact probability in mammals was most likely attributable to their relatively longer chromosomes.

We next asked whether the relatively low frequency of long-range contacts in mammals reflected more stretched CTs compared to those in birds and fish. Using in silico modeling [30], we found that the spatial distances predicted between long-range loci were negatively correlated with chromosome length (Fig. 1E, Additional file 2: Fig. S1C). We used VSR^−1^ and VpM^−1^ to evaluate the stretchiness and condensation of CTs, respectively. The stretchiness and condensation are negatively and positively correlated with chromosome length, respectively (Fig. 1F, G). Which indicated that longer chromosomes are less stretched and more condensed compared with these shorter chromosomes. These observations are consistent with previous reports that showed smaller chromosomes are gene-richer and more active [25, 31, 32], indicating more open and stretched CTs. For example, in chickens, chr. 1 (196.20 Mb) is 5.53 and 17.75 times respectively longer than chr. 6 (35.47 Mb) and chr. 18 (11.05 Mb); but the stretchiness for these three chromosomes are 3.85, 4.41, and 4.81, respectively; and the condensation are 71.95, 23.19, and 8.70, respectively (Fig. 1H). These results further supported the possibility that chromosome length generally affects the overall chromosomal architecture across vertebrates, with longer chromosomes having a generally lower frequency of long-range contact, less stretched and more condensed CTs.

### Inter-chromosomal interactome across species

We next asked whether chromosomal length could affect the distribution of CTs in the nucleus. To compare the relative distribution of CTs across species, we generated an inter-chromosomal contact matrix at 500 kb resolution, i.e., ~ 99.32% of bins had at least 1000 high confidence inter-chromosomal reads (*q*-value < 10^–6^), and subsequently constructed bin interaction networks (BINs) for each species [33, 34] (Additional file 2: Fig. S2A). We analyzed the basic network properties (including network variance, clustering coefficient, average degree, and characteristic path length) of 31 fibroblasts and compared these properties to corresponding random perturbations. In total, the network variances were ~ 278.52-fold higher in these observed networks compared with the randomly shuffled controls, suggesting a strongly non-random distribution of CTs. We observed that all BINs exhibited power law degree distribution (slopes ranged from − 0.82 to − 1.59; Additional file 2: Fig. S2B), where the network topology was dominated by a few highly interactive bins, with most bins exhibiting low contact frequency (Additional file 2: Fig. S2C). These results suggested that the overall distribution of CTs was robust to random perturbations [35].

**Fig. 2:**
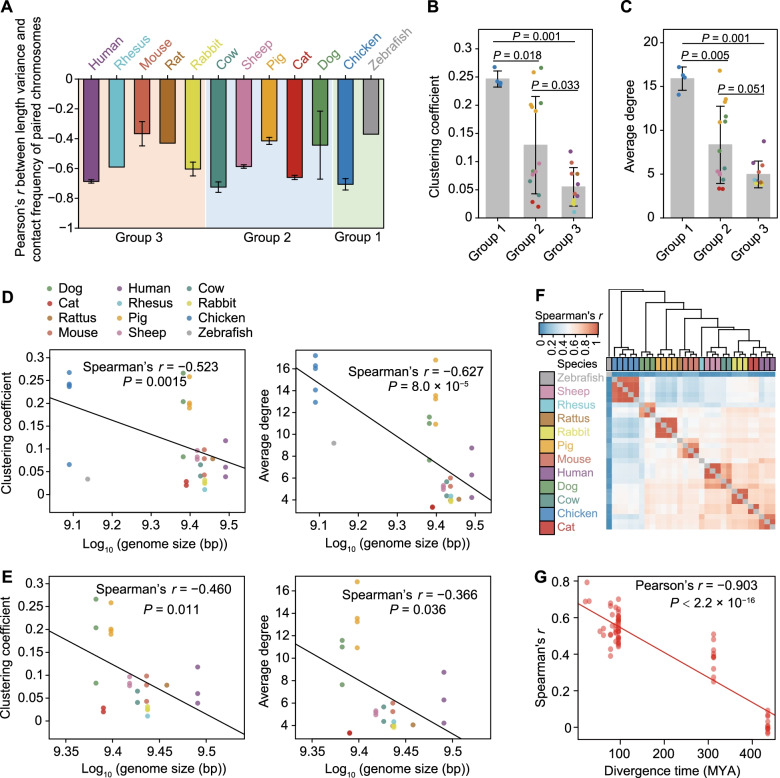
Comparison of the inter-chromosomal interactome across species. **A** Correlation between chromosome length variance and contact probability between each two chromosomes. **B** Differences in clustering coefficients among the three groups of species. **C** Average degree of difference among three groups of species. Two outlier samples (DF1 in chicken and AB.9 in zebrafish) with extremely low clustering coefficients were excluded in (**B**) and (**C**). **D**, **E** Correlation between genome size and two *trans* network parameters (left panel: clustering coefficient, defined as the degree to which the nodes in a network tended to cluster together; right panel: average degree, defined as the average number of neighbors per node) for 12 species (**D**) and 10 mammals (**E**). **F** Inter-species comparison of inter-chromosomal gene-gene contacts. The Spearman’s *r* between contact frequencies of 4,853 single-copy orthologous genes across 12 species. **G** Pearson’s *r* between the inter-species similarity of gene-gene interaction frequency and their divergence time. For (**A–C**), data are presented as mean ± SD. *P* values in (**B**) and (**C**) were calculated using two-sided Wilcoxon rank-sum test

Furthermore, we found that chromosomes with similar lengths were spatially clustered in each sample (Additional file 2: Fig. S3A). That is, the closer the similarity in length between a given pair of chromosomes, the more frequently they interacted with each other, evident in all species examined here by the observation that the probability of contacts between chromosomes was negatively correlated with the differences in their lengths (Pearson’s *r* values are ranged from − 0.18 to − 0.75; Fig. 2A).

We next asked whether substantial divergences occurred between species in their inter-chromosomal contact profile, i.e., how much the chromosomes may contact to each other. To infer the inter-chromosomal contact profiles, we calculated the degree to which the loci in a network tend to cluster together, or clustering coefficient (CC), and the number of neighbors per locus in the network, or average degree (AD). Because the BINs were directly constructed from Hi-C contacts, which were cross-linked at scale by formaldehyde fixation, the CC and AD of contacts should roughly reflect the physical proximity between the contacting chromosomes. Compared to chicken and zebrafish, mammals exhibited relatively weaker inter-chromosomal connectivity (non-mammals in group 1: CC = 0.25, AD = 15.90; mammals in groups 2 and 3 (CC = 0.121 and 0.06, respectively, and AD = 8.08 and 5.03, respectively) (Fig. 2B, C). Given the influence of genome size on the overall genome architecture shown above (Fig. 1C), we next sought to determine if genome size was also associated with these differences in inter-chromosomal contact profiles between species. We found that, indeed, genome sizes were negatively correlated with CC (Spearman’s *r* = –0.523, *P* = 0.0015) and AD (Spearman’s *r* = –0.627, *P* = 8 × 10^–5^) values across 12 vertebrates (Fig. 2D). This negative correlation remained statistically significant even after removal of the huge difference in genome size between mammals and non-mammals by restricting the analysis to ten mammals with comparable genome sizes (CC: Spearman’s *r* = –0.460, *P* = 0.011; AD: Spearman’s *r* = –0.366, *P* = 0.036; Fig. 2E).

To examine whether the inter-chromosomal contacts were functional or not, we scrutinized a subnetwork of the BINs that contained protein coding genes. We then generated GINs (gene interaction networks) based on the propensity of genes to form inter-chromosomal contacts, and then conducted pairwise comparisons between species for orthologous genes. The hierarchical clustering tree based on the similarities identified in these subnetworks roughly mimicked the phylogenetic tree reconstructed for the 12 species (Fig. 1A) and also showed that evolutionarily close species shared a similar propensity for gene-gene contact (Pearson’s *r* = − 0.903, *P* < 2.2 × 10^–16^; Fig. 2F-G). Similar results were found by restricting the analysis to 11 species or 10 mammals (Additional file 2: Fig. S3B, C).

### The local spatial context is largely conserved in ten mammals

**Fig. 3:**
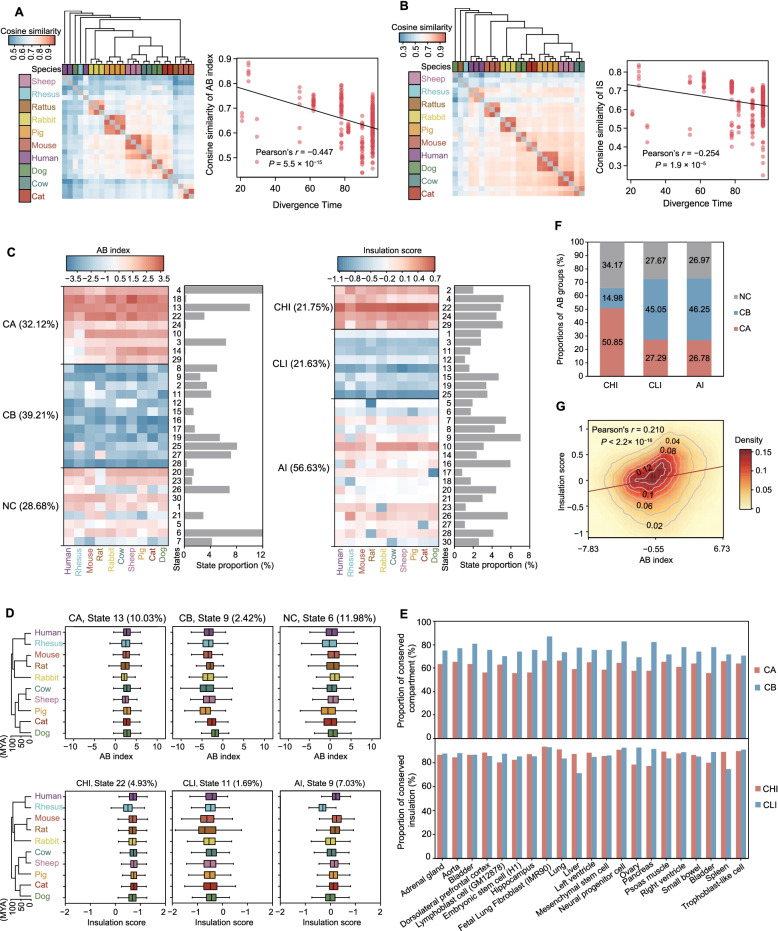
Mammalian A/B compartment phylogenies. **A**, **B** Left panels show Hierarchical Clustering Trees of AB index (**A**) and IS (**B**) for all aligned bins based on cosine similarity of 10 mammals, respectively; right panels represent the Pearson’s correlation of similarity in AB index (**A**) and IS (**B**) for each pair of mammalian species and their divergence time, respectively. **C** The conserved compartment and insulation groups were clustered using cosine similarity (*R* ≥ 0.85 for any two states in the same group). Thirty states could be grouped into conserved compartment A (CA) and conserved compartment B (CB), the remaining states were classified as not conserved (NC) (left panel). Conserved high IS (CHI), conserved low IS (CLI), and ambiguous IS (AI) were also classified (right panel). The percentage of each state is shown on the right. Red and blue indicate positive or negative values, respectively. **D** Examples of AB index (state 9: CB; state 13: CA; state 6: NC) and IS (state 22: CLI; state 11: CHI; state 5: AI) distributions for states with different patterns of conservation. The internal line indicates the median, the box limits indicate the upper and lower quartiles and the whiskers extend to 1.5 IQR from the quartiles. **E** Comparison of predicted patterns of compartment (top panel) and insulation (bottom panel) conservation with correlated regions identified across 21 human tissues or cell lines. **F** The percentage of CA, CB and NC in each IS group. The CHI group harbors more bins with CA (50.85%) than CB (14.98%) states. **G** Correlation between AB index and Insulation Score (IS) for 84,974 bins aligned across the 10 mammalian species

We observed that known features of the A and B compartments in all species, at 20 kb resolution (Additional file 2: Figs. S4A), provided an opportunity to explore the evolutionary patterns of compartmentalization. Given that only a minute proportion of the human genome (for reference) is homologous with those of the 11 (2.07%; excluding zebrafish) and 12 (0.31%) total species, we instead focused on regions of the human genome that shared homology with the ten mammals (27.44%). To this end, we separated the mammalian homologous regions into 84,974 bins, of which 6.52% (or 5,541 bins) carried at least one protein coding gene (hereafter referred to as “gene bins”). The overall similarity of the A/B compartments (Fig. 3A, Additional file 2: Fig. S4B), and the insulation scores (IS) (Fig. 3B, Additional file 2: Fig. S4C) among mammals largely agreed with relationships illustrated by their phylogenetic tree, as well as that of the single copy orthologous gene expression-based tree (Additional file 2: Fig. S4D). As expected, we observed a negative correlation between divergence time and similarities of AB index (Fig. 3A, Additional file 2: Fig. S4B) and IS (Fig. 3B, Additional file 2: Fig. S4C), which thus suggested that evolutionarily closer species shared higher similarity in their patterns of local spatial context. As we only studied the conserved portions of each genome, our conclusions are strictly limited to these regions (from 25.97% of rattus to 32.51% of pig).

We next identified the local regions with conserved or species-specific states that exhibited differences in spatial context and subsequently estimated the probability of divergence in AB index and IS across the ten mammals using the phylogenetic hidden Markov Gaussian processes (Phylo-HMGP) model [36]. We classified the 30 states (determined by *K*-means clustering; Additional file 2: Fig. S5A, B) into three groups based on their AB indexes, i.e., conserved compartment A (CA), conserved compartment B (CB), and non-conserved compartment (NC). Similarly, the 30 states were also classified into three groups based on IS, i.e., conserved high IS (CHI), conserved low IS (CLI) and ambiguous IS (AI) (Fig. 3C, D). We found that the majority of homologous regions (849.74 Mb, 27.44% of human genome) had conserved compartment states across the ten mammals (CA: 272.99 Mb, 32.12%; CB: 333.02 Mb, 39.21%), and only 243.73 Mb (~ 28.68%) were assigned to the NC group. In addition, nearly half of these regions had conserved insulation score states (CHI: 184.80 Mb, or 21.75%; CLI: 183.77 Mb, or 21.63%) (Fig. 3C).

Strikingly, those regions with evolutionarily conserved compartments and IS were also prone to maintain their compartment and IS states in other tissue or cell types. Using publicly available Hi-C data from human subjects [37], we found that ~ 56–67% and 69–87% of the bins with respectively conserved A and B compartments in human fibroblast cells retained the same compartment status in 14 other tissues or 7 other cell types (Fig. 3E), compared with randomized controls (~ 41–52% and 48–58% of the bins with conserved A and B compartments; *P* < 3.7 × 10^–12^, Wilcoxon rank sum test). Similar values of ~ 77–93% were observed for CHI and 65–79% for CLI (Fig. 3E), compared with the randomized controls (~ 52–60% for CHI and 39–48% for CLI; *P* < 3.7 × 10^–12^, Wilcoxon rank sum test).

Notably, the bins with conserved A and B compartment status were found prone to have high and low ISs, respectively (Fig. 3F), leading to a moderate correlation between IS and AB indexes (Pearson’s *r* = 0.21, *P* < 2.2 × 10^–16^; Fig. 3G, Additional file 2: Fig. S5C, D). These findings implied a certain level of co-occurrence between closed and insulated chromatin architecture.

Last, we asked whether genes embedded in the NC regions were enriched with any species-specific functions. Using Metascape [38], we identified several interesting functional groups (Additional file 2: Figs. S6 and S7). For example, genes embedded in human-specific compartment B regions (Phylo-HMGP state 7) were mainly involved in the processes of “development of male gonad and sensory organ” and “renin secretion,” whereas genes embedded in the rat- and mouse-specific compartment A regions (Phylo-HMGP state 20) were mainly involved in the processes of “response to chemokine” and “oxidoreductase activity”. Moreover, genes embedded in the pig-specific compartment A regions (Phylo-HMGP state 26) were mainly involved in the processes of “metalloexopeptidase activity” and “regulation of cellular response to stress” (Additional file 2: Fig. S6A). These results suggested that regions with species-specific evolutionary patterns may contain genes with distinct functions corresponding to phenotypic diversity across species.

### The TADs are evolutionarily conserved gene expression regulatory units

**Fig. 4:**
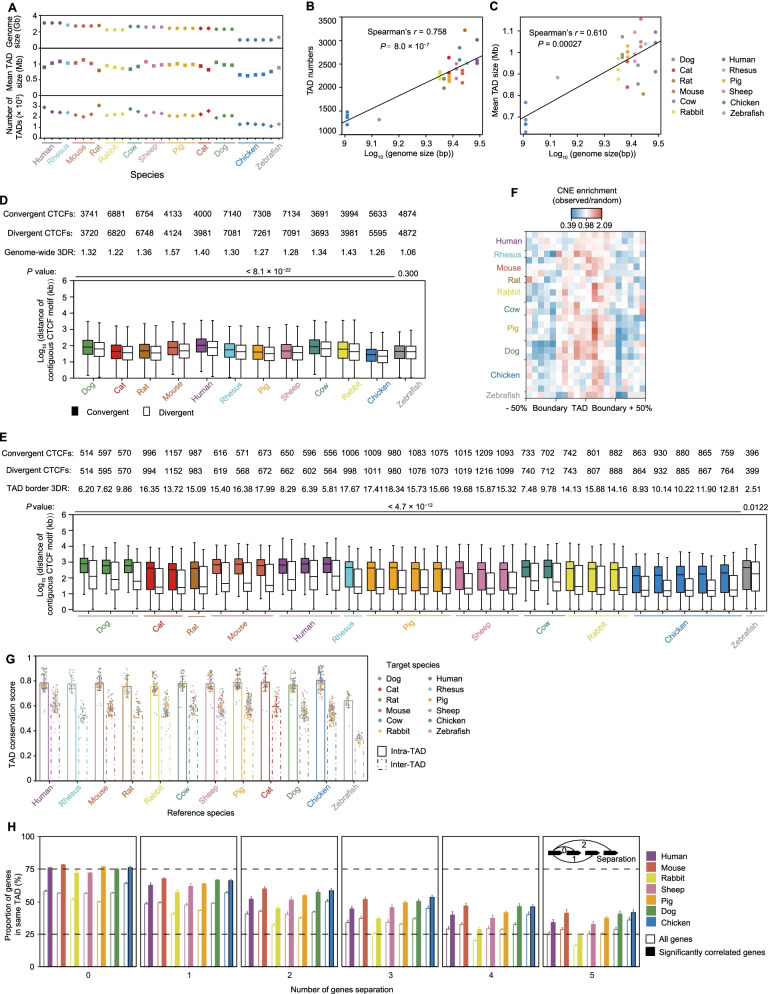
TADs are conserved units of genome organization. **A** Genome size (top panel), average length (middle panel) and number of TADs (bottom panel) in 12 species. **B**, **C** Spearman’s correlation between genome size and TAD number (**B**) or TAD size (**C**) in 12 species. **D** 3DR values computed from the 12 genome assemblies. The colored and white boxes represent “convergent ‘→ ←’ and “divergent ‘← →’” contiguous CTCF motifs. **E** 3DR values of 12 species when accounting for TAD borders. TAD borders were defined as the extended 20 kb regions around the center of each TAD boundary (on each side, 40 kb in total). We only kept CTCF motifs belonging to TAD borders when calculating the 3DR values in the TAD borders. **F** Distribution of CNEs (conserved non-coding elements) in TADs. **G** Plot showing the mean intra- and inter-TAD conservation scores in pairwise comparisons between species. **H** Barplot of the percentage of correlated gene pairs (Pearson’s *r* > 0.9) (slashed color bars) within the same TADs compared to all gene pairs (blank color bars) based on expression data of fibroblasts from 7 species with at least 3 replicates. The tested gene pairs were stratified based on the number of genes by which they were spatially separated. Distances are indicated underneath the barplot. For (**D**) and (**E**), the internal line indicates the median, the box limits indicate the upper and lower quartiles and the whiskers extend to 1.5 IQR from the quartiles. *P* values were calculated using two-sided Wilcoxon rank-sum test. For (**G**) and (**H**), data are presented as mean ± SD

To further explore conservation of TAD profiles across species, we partitioned the genomes of 12 vertebrates into topologically associating domains (TADs) at 20 kb resolution using the directionality index (DI) algorithm in DomainCaller [7] (Additional file 2: Fig. S8A). We observed known TAD boundary signatures around our boundary calls for each species, such as enrichment for transcription start sites (TSSs) of protein coding genes (especially highly transcribed housekeeping genes) at the center of their boundaries in mammals, chickens, and zebrafish (Additional file 2: Fig. S8B). The larger mammalian genomes had more and longer TADs (Fig. 4A–C). Regarding the molecular mechanisms leading to the conservation of 3D genome organization, we examined whether the binding sites of CTCF underwent selection during vertebrate evolution. By scanning the consensus binding motif of CTCF, we found the 3DR values [39] in all 12 vertebrate species are significantly higher than 1 (Fig. 4D). Interestingly, the lowest observed 3DR value was found in the GC-poor zebrafish genome (GC content is 36.54% compared to the highest value of 46.76% found in chicken) [39]. This suggests GC content might contribute to loop formation. We then restrict this analysis to the TAD borders, and found strikingly high 3DR values (ranging from 2.51 in zebrafish to 19.67 in sheep) in all 12 species (Fig. 4E). These results suggest that convergent CTCF poses a strong evolutionary constraint to 3D genome organization that is encoded in the genome. In addition, both TAD similarity and 3DR results (Additional file 2: Fig. S8C, D) indicate genome version has a limited effect on 3D genome organization analyses, as previously suggested [39].

To explore whether TADs were evolutionarily conserved in gene expression regulation, we examined the distribution of high confidence (phastCons ≥0.95) and extended (> 200 bp) conserved non-coding elements (CNEs) as phylogenetic markers around the TADs. The results of this analysis showed that CNEs were enriched within the TADs but depleted at TAD boundaries (Fig. 4F). Moreover, the depletion of CNEs was asymmetric in regions flanking the boundaries, and the highest degree of CNE depletion was observed in boundary regions compared with the interior TAD regions (0.867 average CNEs per bin in boundary regions and 1.132 average within TADs). We also compared the linear positions of single copy orthologous genes between species [40] and found that intra-TAD conservation was stronger than inter-TAD conservation (Fig. 4G), which suggested an evolutionary restriction of intra-TAD co-localization for these genes. We next asked whether the conservation of TAD structure had any functional implications. As expected, these correlated co-expressed gene pairs were significantly enriched in intra-TADs (Fig. 4H). The restrictive influence of TAD structure on gene regulation may be a conserved evolutionary feature.

**Fig. 5:**
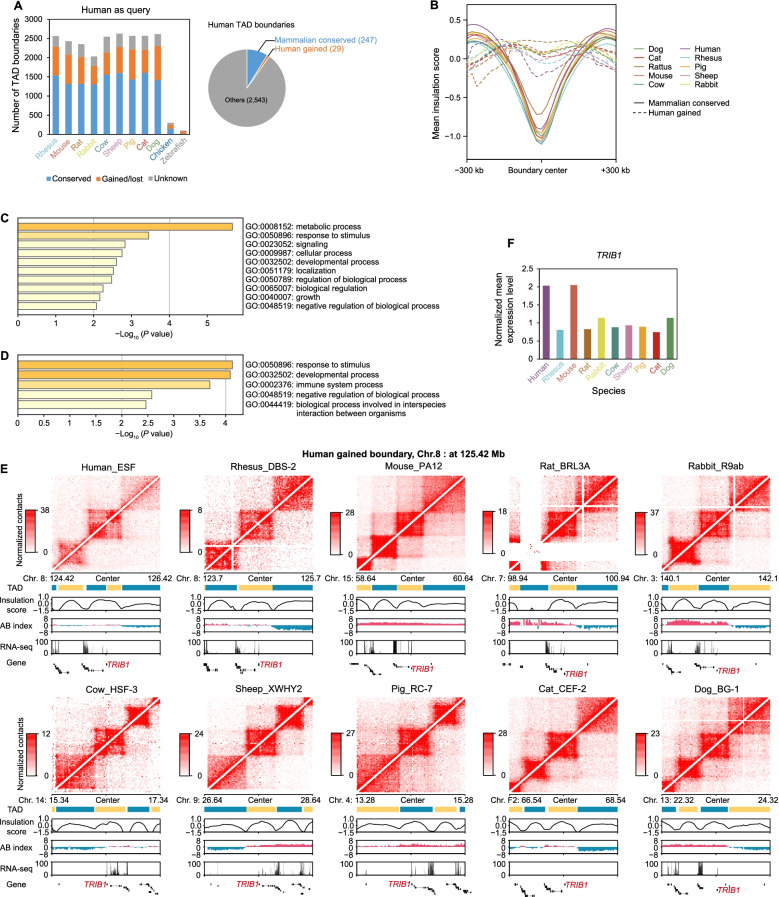
TAD gain and loss. **A** Proportion of conserved and gained/lost TAD boundaries in each target genome (left panel). “Human as query” indicates the human TAD boundaries that were taken as query using the UCSC liftover tool compared to other genomes. The TAD boundaries classified as conserved, gained/lost, or unknown, were determined by the distance to the nearest TAD boundary in the reference genome (conserved: < 40 kb, or 2 bins; non-conserved: > 100 kb, or 5 bins; otherwise, unknown). The number of mammalian conserved and human gained boundaries (right panel). **B** The average insulation score profiles in ±300 kb regions around the mammalian conserved and human gained TAD boundaries. **C** GO enriched terms for genes in conserved human TAD boundary regions. **D** GO enriched terms for genes in gained TAD boundary regions in the human genome. **E** Hi-C contact maps of human gained TAD boundaries correlate with the single-copy orthologue gene *TRIB1* in 10 mammal species. Gene ID of *TRIB1* in each species was marked in red. **F** Normalized mean expression levels of the gene *TRIB1* in 10 mammal species

Luo et al. (2021) reported ~ 55% (*n* = 1513) of the TAD boundaries were conserved across human, rhesus, and mouse, with ~ 14% (*n* = 385) of the TAD boundaries being specifically gained in humans [41]. Using the same definition, we identified conserved and gained/lost TAD boundaries in this study. Briefly, a TAD boundary was considered lost in species A if the region could be “liftovered” and aligned from human to most species but not to species A. A TAD boundary was consider gained in species A if it was not found in any other species. This allowed us to identify 247 conserved and 29 human gained TAD boundaries across 10 mammal species (Fig. 5A). We compared the insulation scores (IS) in conserved and gained/lost TAD boundaries. As a result, we found the conserved TAD boundaries had stronger insulation than those that were gained (Fig. 5B), indicating the stability of conserved TADs during evolution. We performed a Gene Ontology (GO) enrichment analysis in each of TAD category, and found genes in the mammalian conserved TAD boundaries are involved in the metabolic process and growth, in particular the regulation of responses to DNA damage (Fig. 5C). In contrast, TAD boundaries gained in humans were associated with the immune system (Fig. 5D). One example is the human gained TAD boundary containing the *TRIB1*, a gene that is involved in leukocyte differentiation and response to bacterium (Fig. 5E). This implies a close association to human tumor and cellular immune response [42]. As a blood and tissue biomarker of chronic antibody-mediated rejection, this gene plays a key role in transplantation [43]. We found an increased expression in humans than in most other mammal species considered, with the exception of mouse (Fig. 5F). Another example is the human gained TAD boundary containing the *GPM6A*, a gene that is involved in neuronal differentiation and migration of neuronal stem cells [44] (Additional file 2: Fig. S9A). We also showed a primate gained TAD boundary containing the gene *GRM5*, a promising target for the treatment of cognitive deficits in schizophrenia [45], which may be involved in the regulation of neural network activity and synaptic plasticity [46] (Additional file 2: Fig. S9B).

### Some TEs appear highly correlated with chromatin interactions

**Fig. 6:**
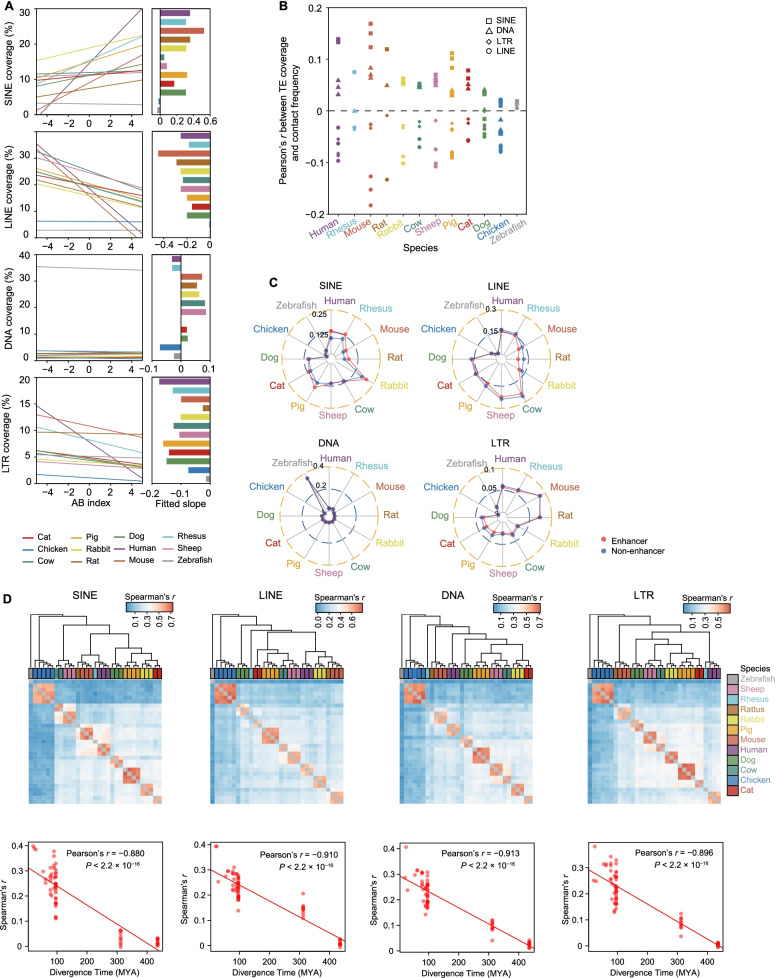
TE and Chromatin architecture in the 12 vertebrate genomes. **A** Correlation between AB index and TE coverage for 12 species. The species associated with the fitted slope are labeled on the right. **B** Correlation between proportion of four TEs and chromatin contact frequency. **C** Enrichment of four TE types in enhancer and non-enhancer (all the other regions) regions of the genome in each species. **D** Spearman’s correlation of TE coverage in enhancer regions among 12 species (upper panels); and correlation between similarity of TE coverage in enhancer regions and evolutionary divergence time in 12 vertebrates (bottom panels)

Transposable elements (TEs) exhibited high diversified proportion among zebrafish, chickens, and mammals [47]. We found that TEs comprised ~ 36.32–47.92% of the assembled genomes of 12 vertebrates (Additional file 2: Fig. S10A, B). We subsequently found that some TE families were either enriched (SINE) or depleted (LINE, DNA, and LTR) in the TAD boundaries (Additional file 2: Fig. S10C). The exceptions were found in cow, sheep, and non-mammalian genomes, although this finding may be attributable to poor genome assembly [48]. Then, we analyzed the correlation between TE coverage and AB index for TE families, and found rather weak, but significantly positive and negative respective correlations for SINE and LINE, respectively (Fig. 6A, Additional file 1: Table S5). This result was in agreement with a recent imaging study which showed that LINE elements are more enriched in heterochromatin in mice [49].

To further explore the potential roles of TEs in chromatin organization across species, we compared the distribution of intra-TAD contact frequencies for the four major TE families. The results revealed a clear trend in mammals in which the proportion of SINE and DNA transposons were positively correlated with the contact frequency, while the LTR and LINE proportion showed a negative relationship (Fig. 6B). However, it warrants mention that these correlations were weak. By contrast, in the chicken genome, the DNA transposons were found to be negatively correlated with contact frequency; in zebrafish, all TE families examined here showed a positive correlation with contact frequency.

Given the limited number of non-mammalian samples in this study, TE distribution merits further investigation in the non-mammals. Furthermore, we found that the degree of correlations with contact frequency was almost always higher for SINEs and LINEs than for DNA transposons and LTRs in all 10 mammals (Fig. 6B), which suggested that the SINEs and LINEs provided a greater contribution to genome organization than the other TE families. Indeed, comparisons of TE family enrichment revealed that SINEs were consistently enriched in enhancer regions for mammals, whereas LINEs were consistently depleted (Fig. 6C). Notably, we were unable to detect any consistent patterns for DNA transposons and LTRs in mammals, nor any patterns for any of the TE families in non-mammals (Fig. 6C). Last, we found that evolutionarily close species shared a higher similarity in their TE coverage in the enhancer regions of these single-copy orthologous genes in the 10, 11 and 12 species (Fig. 6D, Additional file 2: Fig. S10D).

## Discussion

In the present work, we found that genome size is the dominant characteristic that governs differences in the overall spatial conformation of chromosomes between species. It was reported previously that the short and long chromosomes are spatially distributed differently throughout the nucleus [50]. A recent study discovered that genome architecture can be classified into two categories (Type-I includes the three Rabl-like features; and Type-II includes only chromosome territories) across the tree of life [51]. The 12 vertebrates’ genome architectures in this study can be classified to the type-II, and we discussed more detailed patterns of genome organization (especially compartment and TAD). Our findings highlighted the biophysical dynamics governing the physical properties of the nucleus and indicated some universality or conservation of these properties across vertebrates. In line with these findings, genome size has previously been associated with the size of the cell and nucleus [52]. Thus, when dealing with chromosomal macro features, such as CT, extensive caution and rigor are necessary for accurate data interpretation.

Previous studies have suggested that the overall arrangement of CTs is a conserved feature, with short and long chromosomes prone to localization in the inner nucleus and periphery of nucleus, respectively [31, 53]. In our study, we extended this evolutionarily ubiquitous feature by showing that the main characteristics of inter-chromosomal contacts are also largely conserved across vertebrate 3D genomes. The similar gene-gene contact profile in closely related species indicating a general spacial structural foundation for gene co-regulation in vertebrates [54–56]. However, substantial divergence in specific inter-chromosomal contacts occurred at the species level, implying species-specific genomic reorganization. As many studies have shown that the genome is subject to modularization by functional constrains [57–61], future study will help determine how this modularization process may be associated with species level reorganization of inter-chromosomal arrangement during the evolution.

It has already been reported that TAD boundaries are evolutionarily conserved [7, 9, 16–19, 22]. Consider the histone sequences and combination of histone modifications are conserved across many species [62], how epigenetic features involve in 3D genome organization is also worthy of attention. Consistent with CTCF and H3K4me1 were detected as the first and second informative histone marks for predicting TAD boundaries [63], our study also showed a strikingly high 3DR values of CTCF for TAD borders in all 12 species. Furthermore, chromatin domains with high contact frequencies were found correlate with histone marks associated with inactive chromatin such as H3K9me3 and H4K20me3 [62]. H3K4me3-rich regions are associated with active chromatin in an open conformation [62]. In addition, breakpoints enriched at TAD boundaries from mammals to birds, indicating a strong natural selection pressure on maintaining regulatory domain integrity [9, 16, 64]. Avian genome is approximately 3-times smaller than mammalian, consistently fewer number of TADs were observed. This might be caused by less SINE and LINE elements, which has been shown tightly linked to genome architecture [65] presented in avian genomes (5.94–6.24 % in chicken and fish versus 24.84–39.57% in mammals). However, the underlying mechanism of which effect shall be exported in the further. Moreover, due to the difficulty of defining conserved TAD structures and the limited number of orthologous genomic regions across vertebrates, the processes of TAD birth and death have not yet been explored. The increased availability of ancestral genomes and their spatial architecture will facilitate the resolution of some complicated research topics, such as whether large, ancestral TADs were split into smaller domains during the evolution of mammals or if some TADs were lost during avian and fish evolution [66]. Thus, the internal structures of TADs, i.e., sub-TADs and loop webs, warrant further, refined investigation. In total, this study provides Hi-C data for 12 species (including fish, chickens, and mammals), which can serve as a valuable resource to establish a standard for inferring TADs, or for assessment of the evolutionary conservation of TADs across species.

The finding that some TEs are enriched (SINE) or depleted (LINE, DNA, and LTR) in TAD boundaries provides an indication of the roles of TEs in TAD organization, which was largely consistent with recently published imaging data in mice [49]. Notably, the relatively weaker correlations we detected in chicken could be at least partially explained by the smaller proportion of TEs in the chicken genome, suggesting a different potential mechanism for the formation of closed or open chromatin structures. Although, the generic trends we reported here hinted at significant roles that SINEs and LINEs may play in the evolution of chromatin structure, the detailed evolutionary dynamics that the TEs may contribute to determining chromatin architecture remains to be explored. This study may thus serve as a starting point for addressing several questions that were only initially touched upon in our analyses. For example, it remains unclear activity by TEs may alter TADs, and in turn affect regulatory circuits in recent evolutionary and developmental advances. Furthermore, what phenotypic consequences may result from those hypothetical TE-mediated alterations to TADs? Large scale population level data is thus needed to further explore these genomic evolutionary questions.

In addition, there are still some methodological and analytical limitations in this study. First, the Hi-C data were generated by dilution Hi-C, which contains more noise and lower resolution than in situ Hi-C. Second, we have not used the most recent updates for some genome assemblies due to limited time and resources. However, we note that this is highly unlikely to substantially alter our main results and conclusions. Third, the discussed TAD boundary gain and loss only addressed pairwise comparisons between humans and other mammal species. Hence, further comprehensive evolutionary analysis in the future should be implemented with novel methods.

## Conclusions

Here, we compare the 3D genome architecture of 12 vertebrates, including 10 mammals from four distinct lineages, and uncover organizational features that appear to determine the conservation and transcriptional regulation of functional genes across species. We identified a correlation between genome size/chromosome length and long-distance contacts. Genome size and chromosome length were found to be affecting factors in the organization of chromosomal territories (CTs) in the upper hierarchy of genome architecture. Lower hierarchical features, including local transcriptional availability of DNA, were revealed to be selected through speciation processes. The conservation of topologically associating domains (TADs) appears strongly associated with the modularity of expression profiles across species. LINE and SINE transposable elements likely contribute to heterochromatin and euchromatin organization, respectively, during the evolution of genome architecture.

## Methods

### Collection of fibroblasts, Hi-C experiments, and data generation

We collected 25 fibroblast lines from 11 vertebrate species, of which 13 are primary cells (derived from the same tissue in different individuals) and 12 are commercial cell lines (Additional file 1: Table S1). All animal protocols were approved by the Institutional Animal Care and Use Committee of the Institute of Animal Genetics and Breeding (protocol number YCS-B20151604). The methods were carried out in accordance with the approved guidelines.

Most of the fibroblasts were grown in Dulbecco's Modified Eagle Medium (DMEM, 11995-065, Gibco) containing 10% Fetal Bovine Serum (FBS, 10099-141, Gibco) and 1× penicillin/streptomycin (P/S, 15140-122, Gibco), incubated at 37 °C in 5% CO_2_. Dilution Hi-C were performed as in previous a study [25]. Briefly, approximately 20 to 25 million cells were cross-linked with 2% formaldehyde for 10 min at room temperature (20–25 °C), and then glycine was used to quench the formaldehyde in a final concentration of 0.25 M at room temperature for 5 min. Subsequently, cross-linked cells were incubated on ice for 15 min. Nuclei were permeabilized by a Dounce homogenizer in the presence of cold lysis buffer (10 mM Tris-HCl, pH 8.0, 10 mM NaCl, 0.2% IGEPAL CA-630, and 1× protease inhibitor solution). DNA was digested with 400 units of HindIII, and the ends of restriction fragments were labeled using biotinylated nucleotides and ligated in a small volume (8 mL). After reversal of cross-links, ligated DNA was purified and prepped for Illumina sequencing.

All paired-end (150 bp in read length) sequence data were generated using Illumina Hiseq X Ten platform by Novogene Bioinformatics Technology Co. Ltd.

### Raw sequence quality checking and filtering (processing raw data to clean data)

We also downloaded the publicly available Hi-C data of four porcine fibroblasts [26] and two mouse fibroblasts [27] (Additional file 1: Table S1) for analysis. Together, we processed Hi-C data of 31 fibroblasts from 12 species. To avoid reads with artificial bias, we removed the following types of reads: reads with ≥ 10% unidentified nucleotides (N); reads with > 10 nt aligned to the adapter, allowing ≤ 10% mismatches; reads with > 50% bases having phred quality < 5. Consequently, after quality checking 4.82 Tb of raw data, we obtained 4.58 Tb of high-quality paired-end reads, including 95.18% and 89.57% nucleotides with phred quality ≥ Q20 (with an accuracy of 99.0%) and ≥ Q30 (with an accuracy of 99.9%), respectively.

### Mapping and filtering of Hi-C reads

Read mapping and filtering of the initial Hi-C data analyses were conducted by Hiclib [28]. The high quality paired-end reads of the 12 species were mapped to their own reference genomes by Bowtie2 [67] (--very--sensitive) through iterative mapping. Mapped reads were filtered using Hiclib with default parameters, discarding the invalid self-ligated and un-ligated fragments, as well as PCR artifacts. To avoid the effects of X chromosome inactivation, subsequent analyses excluded sex chromosomes (i.e., only Hi-C contact statistics contain sex chromosomes). The reference genome assemblies for 12 species included: Human (GRCh38.p10), Rhesus (rheMac8), Mouse (GRCm38.p6), Rat (Rnor6.0), Rabbit (OryCun2.0), Cow (bosTau7), Sheep (oviAri3), Pig (susScr11), Cat (felCat8), Dog (canFam3), Chicken (Gallus_gallus-5.0), and Zebrafish (danRer10) respectively.

### Generation and normalization of contact matrices

We generated contact matrices using varying bin sizes (with 20 kb, 100 kb, 500 kb, and 1 Mb resolution). For example, to calculate the contact matrix, we divided the linear genome into 20 kb bins and counted the number of contacts we observed between each pair of bins. The number of contacts observed between locus *i* and locus *j* is denoted *M*_*ij*._ We normalized the Hi-C matrices as previously described by Imakaev et al. [28]. Normalization reduced the differences in interactions within biological replicates and among samples. Normalization also homogenized interactions in different samples at the same distance without disturbing interaction frequency decay as genomic distance increased. After normalization, we obtained the corrected interaction matrix for each whole genome.

### Correlation among cells in the same species by observed matrix

We calculated the correlations between Hi-C replicates in the same species on a per chromosome basis using HiCRep [68] with 100 kb resolution data. This method calculated correlations by considering two dominant spatial features of Hi-C data: distance dependence and domain structure. The method first smoothed the given Hi-C matrices to help capture domain structures and reduced stochastic noise caused by insufficient sampling. It then addressed the distance-dependence effect by stratifying Hi-C data according to genomic distance.

### Physical models of chromosomes

Physical models of each chromosome in each sample were calculated at 100 kb *cis* and 1 Mb *trans* resolution using miniMDS [30]. Volume and surface areas of the simulated chromosomal territories were calculated using convex hull (https://docs.scipy.org/doc/scipy/reference/generated/scipy.spatial.ConvexHull.html). We first computed volume and superficial area for each chromosome. Volume to surface area ratio (VSR) and volume per Mb in sequence (VpM) were calculated to measure the plasticity of all CTs. For any given volume, the sphere contains the minimal surface while the stretched territory is expected to have the larger surface. Hence, higher VSR^−1^ indicates more stretch. Higher VpM^−1^ indicates more condense chromatin state. The structural models were inferred using multidimensional scaling (MDS) analysis provided in the Python package miniMDS [30] and visualized with PyMOL [69]. Surface reconstructions were performed by Meshlab [70] with the original point cloud data (only the point cloud group with XYZ 3D coordinates) generated by miniMDS.

### Evolutionary conservation of the inter-chromosomal interactome

#### Statistical confidence of inter-chromosomal interactions

We binned the reference genomes into 500 kb segments, which yielded sufficient read coverage for each inter-chromosomal interaction bin pair. As described in Kaufmann et al. [33], we calculated the *p*-values for contact significance for each inter-chromosomal interaction bin pair, and this calculation was performed separately for each pair of chromosomes.

$$P\left({bin}_a,{bin}_b\right)=\sum_{i=k}^n\left(\genfrac{}{}{0pt}{}{n}{i}\right){m}_{norm}^i{\left(1-{m}_{norm}\right)}^{n-i}$$

Here, bin_a_ and bin_b_ are two 500 kb bins, *m*_norm_ is the average interaction probability of all pairs of fragments from bin_a_ and bin_b_ (dividing the product of fragment numbers in bin_a_ and bin_b_ by the product of fragment numbers in the two chromosomes containing bin_a_ and bin_b_). *K* is the number of read pairs representing contacts between bin_a_ and bin_b_, and *n* is the total number of read pairs representing contacts between the two chromosomes containing bin_a_ and bin_b_. The *p*-value indicates the significance of interactions between two inter-chromosomal bins, as the probability that the number of read pairs representing an interaction is larger than (or as high as) our observation, based on the background probability depicting Hi-C biases.

Benjamini and Hochberg-based false discovery rates were further calculated. To remove bias, we also normalized the *q*-values against chromosome length. In brief, a normalization factor was calculated by dividing the product of lengths for the two chromosomes containing bin_a_ and bin_b_ by the product of lengths for the two longest chromosomes. The *q*-values were normalized by multiplying the respective normalization factor.

#### Inter-chromosomal bin interaction networks (BINs) and randomization

We generated binary interaction relationships for 500 kb genome segments based on a *q*-value cutoff, which were further converted into inter-chromosomal bin interaction networks (BINs). In brief, genomic 500 kb bins were considered nodes, while high confidence interaction relationships between pairs of inter-chromosomal segments were regarded as edges (nodes are the bins and the edges are the inter-chromosomal contacts). As described by Witten and Noble [71], we first generated a basic random network by randomly distributing segments in a unicube and extracting edges between the *x* closest pairs based on Euclidean distance, with *x* representing the number of edges observed in the original network. Using the approach described by Kruse et al. [34], we permuted the set of edges (E) 10 x |E| times and then switched edges between neighbors if the change increased transitivity in the random network to better simulate the clustering characteristics of the original network.

#### Basic network properties analysis

We visualized the networks using Cytoscape [72] and employed its analytical tools to calculate the following basic network statistics (network variance, clustering coefficient, average degree, and characteristic path length) as previously described [33]. The network variance is a parameter that measures the variance of the bin interaction network, and its conformity to the Poisson distribution is taken by dividing the variance by the mean.

$${x}_d=\frac{\sum_{i=1}^n{x}_i}{n}$$

$${\mathrm{S}}_d^2=\frac{1}{n-1}{\sum}_{i=1}^n{\left({x}_i-{x}_d\right)}^2$$

$$\mathrm{Nework}\ \mathrm{variance}=\frac{S_d^2}{x_d}$$

where *n* is the number of nodes, *x*_i_ is the degree of each node, *x*_d_ is the mean of degree, *Sd* is the variance of degree. The network variance is close to 1 in randomized networks, and greater than 1 when the network deviates from random expectations [33].

Clustering coefficient indicates the degree to which the nodes in a network tend to cluster together [33], which is calculated for each node as:

$${C}_n=\frac{2{\mathrm{e}}_n}{\left({k}_n\left({k}_n-1\right)\right)}$$

where *n*, *e*_n_, and *k*_n_ represent a node in the network, the number of connected pairs between all neighbors of node *n*, and the number of neighbors of node *n*, respectively.

A network’s clustering coefficient represents the average clustering coefficient of all nodes, where nodes containing less than two neighbors are set to *C*_*n*_ = *0* in order to avoid an overestimation of clustering in the presence of many singletons.

#### Profiling evolutionary dynamics of inter-chromosomal contacts

For each 500 kb genomic bin, we estimated the inter-chromosomal contact ratio by dividing the inter-chromosomal contacts by the sum of intra- and inter-chromosomal contacts aligned to their respective bins. We further calculated Spearman’s correlations between samples from different species, and summarized the between-species correlations using the average of biological replicates. Moreover, we compared the between-species divergence time and summarized correlation, and estimated their agreement using Pearson’s correlation.

### Identification of A/B compartments

Compartments A/B were identified using both principal component analysis (PCA; eigenvector decomposition) and AB index, as previously described [73]. Briefly, a Pearson correlation matrix at 100 kb resolution was generated using the “cor” function in R. PCA was performed on the correlation matrix using the “prcomp” function in R. The first 3 PCs were then obtained. Bins at 100 kb resolution with a positive Spearman’s correlation between PC1 values and gene density were defined as compartment A (at 100 kb resolution). The other bins were classified as compartment B (at 100 kb resolution). Subsequently, the AB index was calculated at 20 kb resolution, which represents the relative likelihood of a sequence interacting with either A or B defined at 100 kb resolution. The 20 kb bins with positive AB index values (more association with A) were classified as compartment A, while those with negative AB index values (more association with B) were classified as compartment B.

### RNA-sequencing

Total RNA was isolated from fibroblasts using the standard TRIzol method (Invitrogen). RNA-seq libraries were sequenced on an Illumina HiSeq X Ten. Paired-end reads were aligned to reference genomes using STAR (2.6.0c) [74]. Transcript quantification was conducted with kallisto (0.44.0) [75].

We first used gene expression values estimated by RNA-seq in the twelve different vertebrate species. We restricted the analyses to the set of 7846/6322/4881 protein-coding genes that could be identified as single-copy orthologous genes across the 10/11/12 species, respectively, and used log_2_-transformed expression values (TPM + 1) for genes with > 0.5 TPM. For across species RNA-Seq data comparison, we normalized these expression values in the way described in a previous study [76].

### Characteristics (GC content, RNA expression, and proportion of TEs) for compartments A/B

We divided the genomes of each species into 20 kb compartment A/B bins according to the AB index values of each bin, then calculated the GC content of each bin, and calculated the proportion of each TE type in each 20 kb bin based on the four classified TE types (SINE, LINE, DNA, and LTR). For the levels of gene expression of each individual in the 20 kb window, for a single gene in the 20 kb window (i.e., the TSS was in this bin), the log_2_(TPM + 1) of this gene was used as the expression level for this 20 kb bin; if the 20 kb bin contained multiple genes, the after log_2_(average TPM + 1) of these genes was used as the expression level for this 20 kb bin.

### Continuous-trait probabilistic model for comparing multi-species AB index and insulation score data

To obtain AB index values in the orthologous genome regions across the ten mammalian species, we collected the AB index and insulation score values for each 10 kb bin of the human genome and its orthologous regions in each of the other species. First, the genome assemblies (Human (GRCm38.p3), Mouse (GRCm38.p4), Rat (Rnor6.0), Rabbit (OryCun2.0), Rhesus (MMUL_1.0), Cow (UMD3.1), Sheep (Oar_v3.1), Pig (sscrofa10.2), Cat (Felis_catus_6.2), and Dog (CanFam3.1)) were downloaded from the UCSC genome browser (http://genome.ucsc.edu/). Second, we used the human genome (GRCm38.p3) as the reference and divided the reference genome into 10 kb bins. We then aligned each bin in the human genome to each of the other species with reciprocal mapping using liftOver (https://genome.ucsc.edu/cgi-bin/hgLiftOver) to obtain the orthologous regions.

We further applied Phylo-HMGP [36], a probabilistic model-based approach for phylogenetic hidden Markov Gaussian processes, that enables classification of the genomic regions into a predefined number of states, taking into consideration both spatial dependencies along the entire genome as well as temporal dependencies across species in the phylogeny.

In order to confirm conservation of compartment A/B and insulation condition in other tissues and cell lines, we downloaded human Hi-C data [37] obtained from 14 tissues and 7 cell types (GSE87112). We first calculated a consistent compartment A/B or IS ratio in these 21 tissues or cell types, compared with the previously classified Conserved A/B compartments (CA and CB) or IS. Second, we randomly assigned the true A/B indices or insulation scores of these 21 tissues or cell types to the whole genome; then, we also calculated a consistent compartment A/B or IS ratio. Wilcoxon rank-sum test was used to determine the significance of consistency between our classified conserved compartments or IS and real compartment or IS states in other tissue or cell types.

### Identification of topologically associated domains (TAD)

The normalized contact matrix was used as input to perform TAD identification as previously reported [7]. Directionality index (DI) was calculated to 2 Mb upstream and 2 Mb downstream along the center of each bin at 20 kb resolution and hidden Markov model (HMM) was then used to predict the states of DI for final TAD generation.

We used the same criteria for 400 kb resolution (distance between the two adjacent TADs) to distinguish unorganized chromatin from topological boundaries, that is the topological boundaries were less than 400 kb and the unorganized chromatin was greater than 400 kb [7].

### Transcriptional start site (TSS) enrichment analysis

We downloaded the GTF files of our selected 12 species from the Ensemble Database (http://asia.ensembl.org/index.html) and extracted the TSS information for protein coding genes from GTF files by custom python scripts. For enrichment analysis, we identified the mid-point of each boundary region and calculated the density of transcription factors in 10 kb bins for +/− 500 kb from the mid-point.

A list of human housekeeping genes was downloaded from http://www.tau.ac.il/~elieis/HKG/. To obtain housekeeping genes of other species, we used the gene symbols for human genes to identify the corresponding genes in other species.

### CTCF motif calling and calculation of 3DR

We used the vertebrate CTCF motif position frequency matrix MA0139.1 from the JASPAR database (http://jaspar. genereg.net/). We scanned CTCF binding sites on the following genome assemblies: bosTau8, canFam3, felCat5, galGal5, mm10, oryCun2, danRer10, oviAri3, hg38, rheMac8, rn6, susScr11.1. For this purpose, we used MEME FIMO program with default parameters (http://meme-suite.org/doc/fimo.html). A quantile of 70% was used as a threshold for further analyses.

We estimated the following ratio 3DR:

$$3\mathrm{DR}= median\left({d}_{\to \leftarrow}\right)/ median\left({d}_{\leftarrow \to}\right)$$

that was the ratio of two medians: the median of the distances between two contiguous motifs in convergent orientation (noted “→ ←”) and the median of the distances between two contiguous motifs in divergent orientation (noted “← →”) [39]. A 3DR significantly greater than one reflects CTCF looping in the genome.

### Conserved non-coding element (CNEs) distributions in TADs

The CNE information for our selected 12 species was downloaded from CEGA database (https://cega.ezlab.org/) [77]. We screened the CNEs using phastCons with a cut-off of ≥ 0.95 and length > 200 bp. We then calculated the CNE distributions in TADs as follows: we enlarged TAD regions by 50% of their total length on each side, then subdivided the TAD regions into 20 equal-sized bins and computed the number of overlapping CNEs.

### TAD conservation across 12 species

We introduced conservation scores to quantify the conservation of TADs across species, which were determined by the position of orthologous genes within TADs between species [40]. We used each species genome as a target and other genomes as the query. Only the single copy orthologous genes between any two species were used to compute conservation scores.

If a TAD in the target (reference) genome contained one or more conserved gene pairs, we counted this as intra-TAD conservation. When inter-TAD gene pairs occurred together in the reference species on the same chromosome as in target species, we counted this as inter-TAD conservation. Both intra- and inter-TAD conservation scores were calculated as the percentage of TADs in which they occurred.

### Co-expression of intra-TAD genes

To determine whether genes in the same TAD had a higher probability of co-expression, we calculated the Pearson correlation coefficient for all neighboring genes on a chromosome. We then selected gene pairs that had a correlation coefficient > 0.9 and stratified the gene pairs based on whether they were found in the same TAD or not. We scored the correlation coefficients at different distances, where *d* = 0 representing immediately adjacent genes, *d* = 1 for gene pairs with one gene in between, et cetera. We performed our analysis separately on seven species (human, mouse, rabbit, sheep, pig, dog, and chicken) with at least three biological replicates.

### Compiling TEs, correlation analysis between TEs and other chromatin characters

The coordinates of TEs in each genome of the 12 species were downloaded from the UCSC (http://genome.ucsc.edu) database. Based on the classification, we divided all TEs into four major types (LINE, SINE, LTR, and DNA) and discarded the TEs with uncertain categories. The proportion of certain TE types or TE families was defined as the number of nucleotides in the chromatin interaction bin-pairs using the length and annotation of TEs.

To further explore the role of TEs in distal regulation in vertebrate genomes, we then compiled the Hi-C data and TE annotation for the 31 fibroblasts to conduct correlation analysis. We used the mean frequency of inter-domain CI (chromatin interaction) frequency as the lower threshold for each cell line and discarded all the bin-pairs whose CI frequencies were lower than the threshold in all cell lines (assuming they were non-informative or background noise). We then calculated the number of bin-pairs to be filtered out by setting upstream thresholds, and finally removed the CI frequencies with less than 100 bin-pairs by 1 in successive steps (to ensure there were enough bin pairs to evaluate mean proportion of TE). After applying the upper thresholds in each cell line, the removed bin-pairs only occupied from ~ 0.02% in rhesus to ~ 0.27% in chicken fibroblasts. Finally, we were able to retain ~ 1,044,957 bin-pairs for chicken to ~ 5,112,162 bin-pairs for human.

The Pearson correlation coefficient (PCC) between the proportion of different TE families and the CI frequencies in each bin-pair were calculated. Finally, combining the AB index value of each sample for every 20 kb bin, the PCC between AB index and the proportion of TEs (SINE, LINE, DNA and LTR) was calculated using the ‘cor’ function in R.

### Identification of putative enhancer regions

We identified the putative enhancer regions by PSYCHIC [78]. The 20 kb normalized contact matrix was split into a smaller matrix (20 Mb × 20 Mb) with 10 Mb steps of overlapping length, and we analyzed the resultant matrix in PSYCHIC. Subsequently, overrepresented interactions were identified by PSYCHIC at default parameters with promoter regions. Promoter regions were defined as 2000 bp upstream to 500 bp downstream of the TSS site. When at least one non-promoter region was in either one of the two bins involved in a chromatin interaction and one promoter in the other, this interaction was designated as a putative promoter-enhancer interaction (PEI). The genome coordinates of PEIs in each split were then adjusted into the initial position by housing-scripts. We then kept the high confidence PEIs with FDR values ≤ 0.01 and interaction distance ≥ 20 kb. The non-promoter region in these high confidence PEIs were defined as putative enhancers.

## Supplementary Information

**Additional file 1: Table S1.** Sample information and data quality. **Table S2.** Hi-C data mapping and filtering. **Table S3.** Insert size distribution of *cis* contacts. **Table S4.** Resolution of 31 cell lines. **Table S5.** Correlations between AB index and TE coverage.

**Additional file 2: Figure S1**. Whole genome contact frequency. **Figure S2**. Bin interaction networks. **Figure S3**. Inter-chromosome interactions. **Figure S4**. Mammalian A/B compartment phylogenies. **Figure S5**. Thirty A/B compartment states predicted using Phylo-HMGP. **Figure S6**. Gene functions of species-specific compartments and conserved A compartments. **Figure S7**. Gene functions of conserved B compartments. **Figure S8**. TADs are structural and regulatory units conserved across vertebrates. **Figure S9**. Examples of human gained and primate gained TAD boundaries. **Figure S10**. TEs and genome architecture.

## Data Availability

All data generated or analyzed during this study are included in this published article, its supplementary information files and publicly available repositories. The raw Hi-C and RNA-seq datasets of four pig and two mouse samples were available in the SRA repository (SRA IDs: PRJNA482496 [79], PRJNA338854 [80], and PRJNA345113 [81]). The Hi-C datasets of 14 tissues and 7 cell types were available in the GEO repository (GEO ID: GSE87112 [82]). The raw sequence data and processed data generated in this study are available in the GEO repository (GEO ID: GSE167581 [83]).

## Abbreviations

3D: Three-dimensional
CT: Chromosome territory
TAD: Topologically associating domain
TE: Transposable element
Hi-C: High-throughput chromosome conformation capture
MYA: Million years ago
CC: Clustering coefficient
AD: Average degree
BIN: Bin interaction network
GIN: Gene interaction network
IS: Insulation score
Phylo-HMGP: Phylogenetic hidden Markov Gaussian processes
CA: Conserved compartment A
CB: Conserved compartment B
NC: Non-conserved compartment
CHI: Conserved high insulation score
CLI: Conserved low insulation score
AI: Ambiguous insulation score
CNE: Conserved non-coding element
GO: Gene Ontology
PEI: Promoter-enhancer interaction
DMEM: Dulbecco’s Modified Eagle Medium
FBS: Fetal bovine serum
P/S: Penicillin/streptomycin
VSR: Volume to surface area ratio
VpM: Volume per Mb in sequence
MDS: Multidimensional scaling
PCA: Principal component analysis
DI: Directionality index
HMM: Hidden Markov model
TSS: Transcriptional start site
PCC: Pearson correlation coefficient
CI: Chromatin interaction
