# Supplementary material for: Comparative 3D genome architecture in vertebrates
Source: BMC Biol. 2022 May 6;20:99. doi: 10.1186/s12915-022-01301-7 (PMC9077971; doi:10.1186/s12915-022-01301-7)
Supplement: Supplementary file 2 — Additional file 2: Figure S1. Whole genome contact frequency. Figure S2. Bin interaction networks. Figure S3. Inter-chromosome interactions. Figure S4. Mammalian A/B compartment phylogenies. Figure S5. Thirty A/B compartment states predicted using Phylo-HMGP. Figure S6. Gene functions of species-specific compartments and conserved A compartments. Figure S7. Gene functions of conserved B compartments. Figure S8. TADs are structural and regulatory units conserved across vertebrates. Figure S9. Examples of human gained and primate gained TAD boundaries. Figure S10. TEs and genome architecture. [file 12915_2022_1301_MOESM2_ESM.docx]

**Additional file 2**


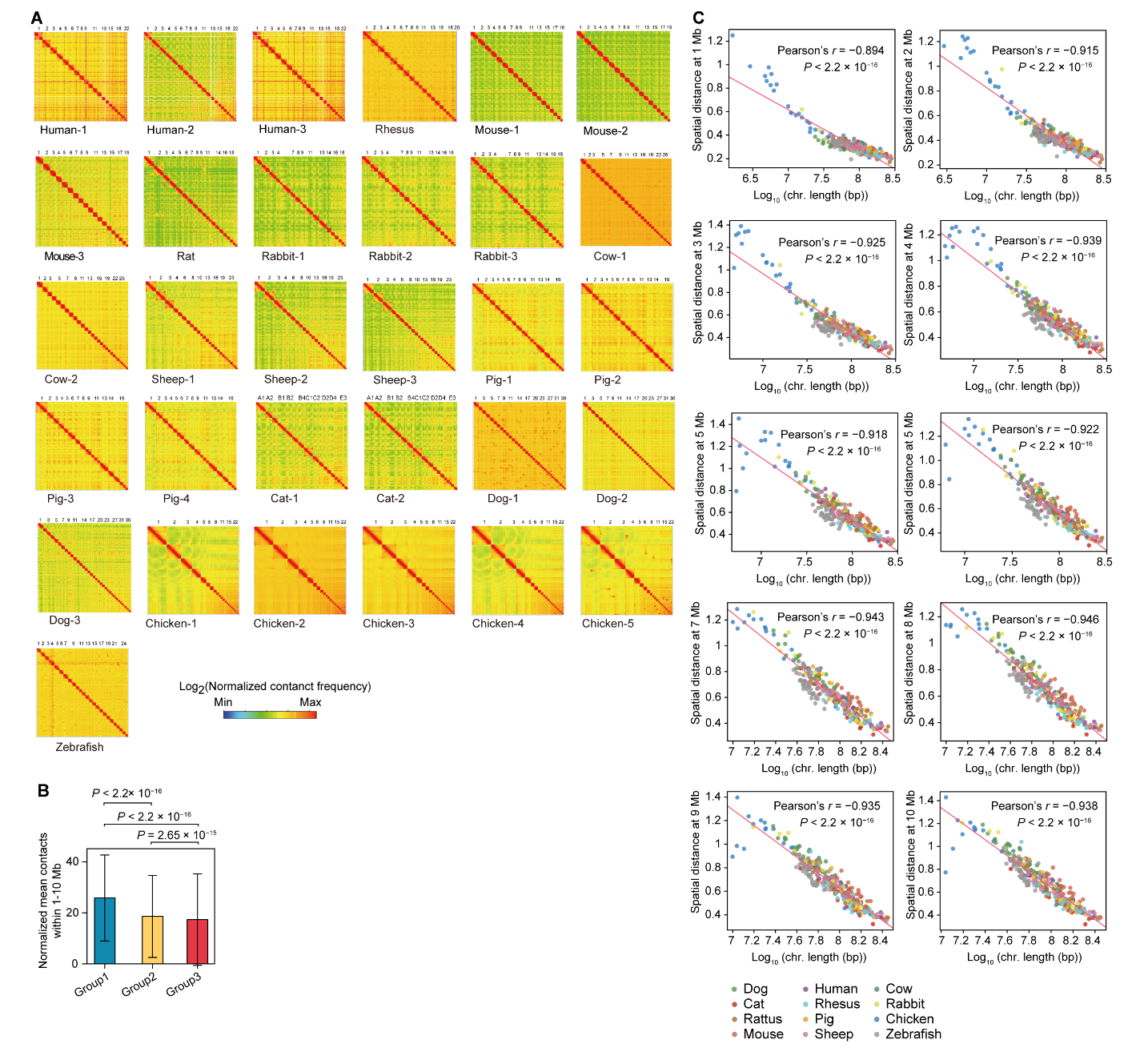


Fig. S1 Whole genome contact frequency. A Whole-genome Hi-C relative contact maps at 1 Mb resolution of 31 samples from 12 species. B Comparison of long-range (*s* from 1 Mb to 10 Mb) contact frequency between three groups. Data are presented as mean ± SD. *P* values were calculated by two-sided Wilcoxon rank-sum test. C Correlation between spatial distance at 1–10 Mb and chromosome length of each two loci.


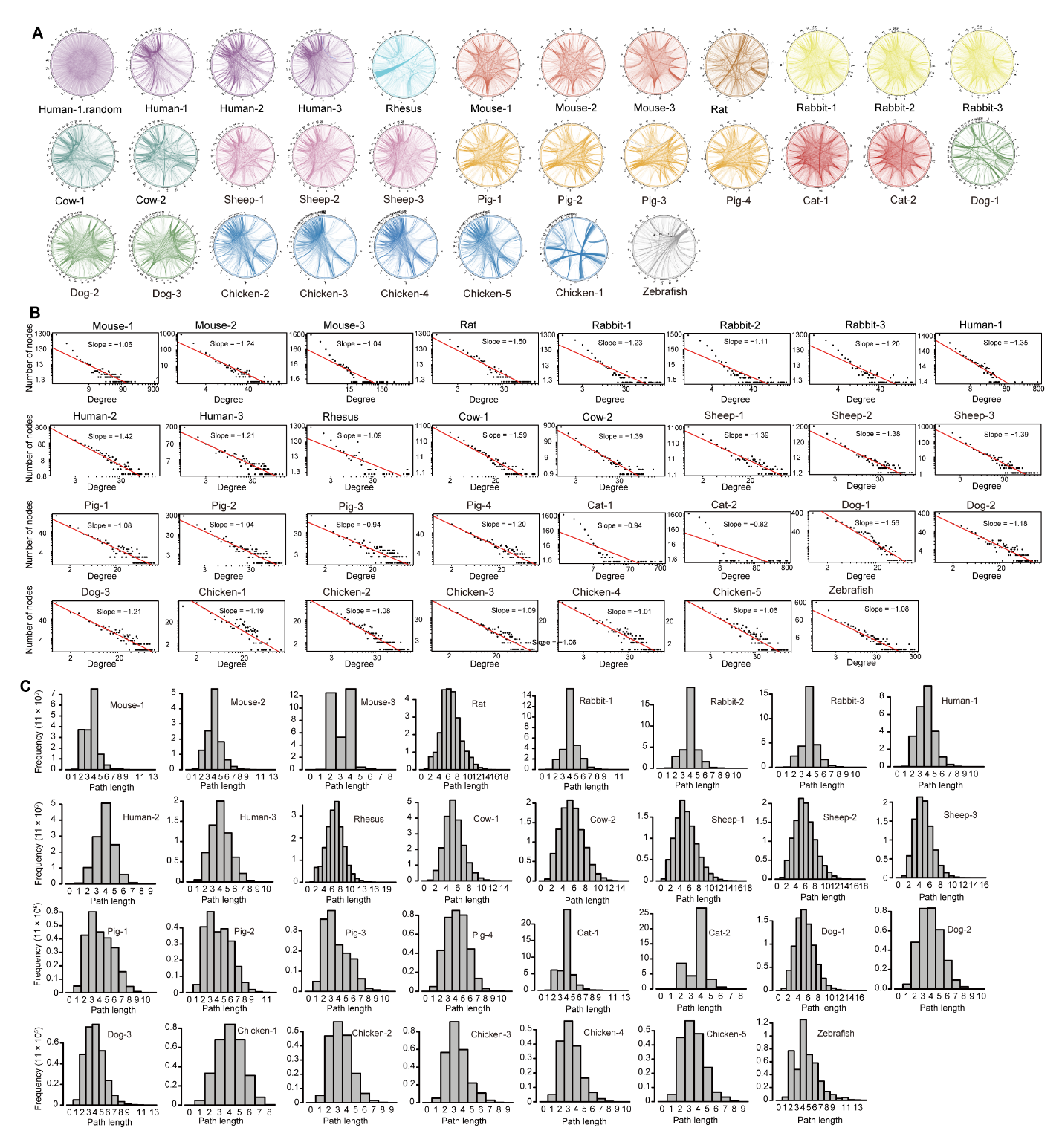


Fig. S2 Bin interaction networks. A Circos visualization of the segment interaction network in one random human sample and 31 samples collected from 12 species with a *q*-value cutoff of 1.0 × 10^−6^. The banded ideograms are chromosomes and the lines between them imply high-confidence contacts. B Distribution of degrees (the number of neighbors per node) for each of the 31 fibroblast bin interaction networks. Degree distribution is shown as a log-log plot. Low degrees are very common, while higher degrees are less frequent. The red line is the fitted power law function with slope −0.82 to −1.59. C Distribution of shortest path lengths for the 31 fibroblast bin interaction networks. Shortest path lengths are normally distributed and center around a medium path length of 4–6, which is mainly caused by the presence the power-law distribution of the number of neighbors per node.


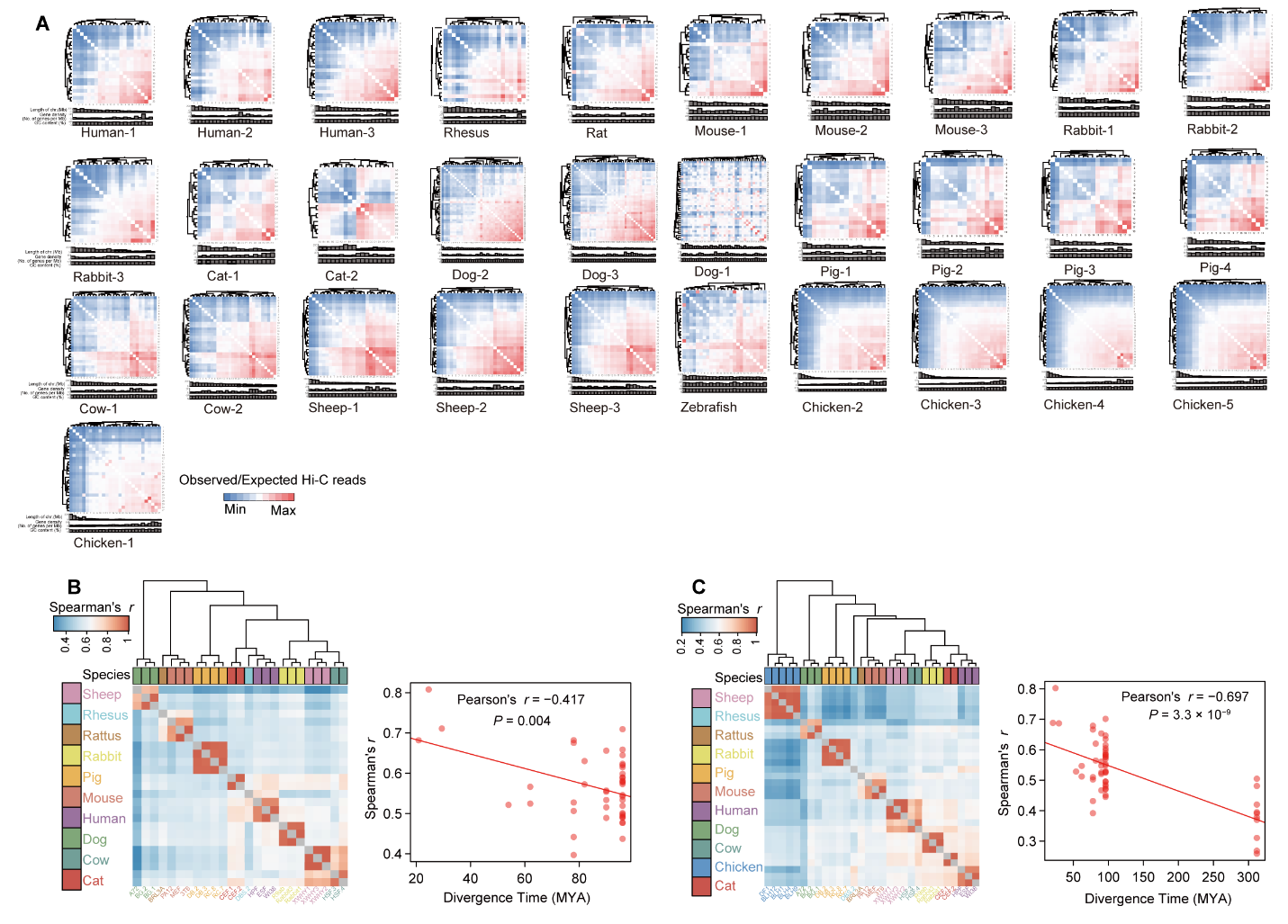


Fig. S3 Inter-chromosome interactions. A Whole-genome contact profiles among chromosomes. The two-dimensional heatmaps show the observed number of interactions between any pair of chromosomes divided by the expected number of interactions between those chromosomes for the fibroblasts of 12 species. Gray histograms indicate chromosome length (Mb), gene density, and GC content of each chromosome. B, C Trans-interactions of orthologous genes. The Spearman correlation of *trans* contact frequency of fibroblasts for 1:1 orthologous genes across 10 mammals (B, left panel) and 11 species (C, left panel). Pearson correlation between the correlation coefficient for *trans* contact frequency of each pair of species and their divergence time in 10 mammals (B, right panel) and 11 species (C, right panel).


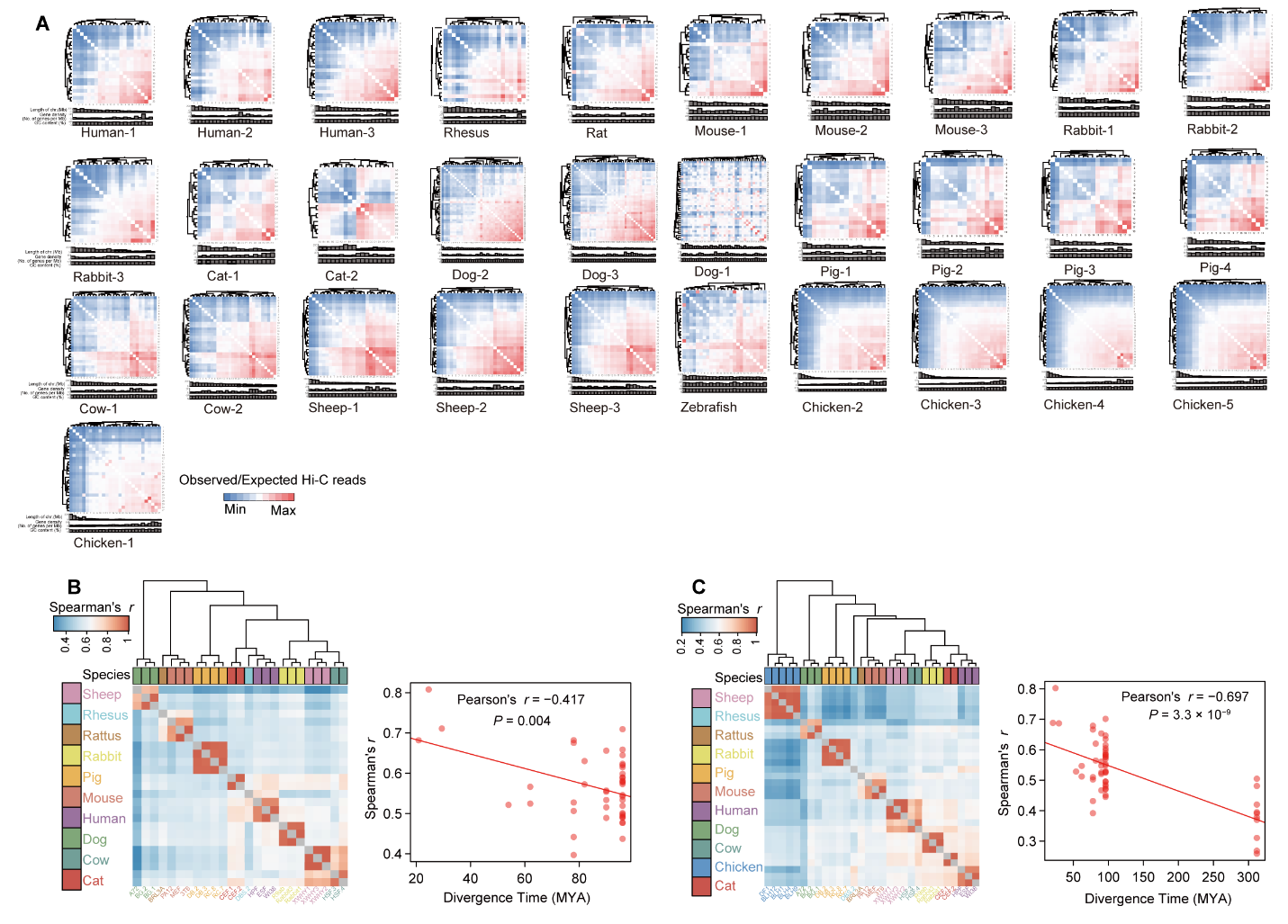


Fig. S4 Mammalian A/B compartment phylogenies. A Genomic features (GC content, gene expression, LINE and SINE proportion, gene density, and isochore proportion) of A/B compartments across 12 species. GC content indicates GC ratio in each 20 kb bin; TE Proportions: base proportions of each TE type in each 20 kb bin; RNA expression: log_2_(average TPM+1) of the genes in each 20 kb bin; Gene density: number of genes per Mb. For the proportion of each type of isochore in compartment A and B, the ‘unknown’ refers to low quality compartments with AB index close to 0. Data are presented as mean values of all bins. B Hierarchical Clustering Tree of AB index values for all gene bins across ten mammals based on the Cosine similarity among them (left panel) and Pearson’s *r* values plot showing the relationship between AB index similarity and divergence time for each pair of mammalian species (right panel). C Hierarchical Clustering Tree of insulation scores (IS) for gene bins across ten mammalian species based on the Cosine similarity among them (left panel) and Pearson’s *r* values plot showing the relationship between IS similarity and divergence time for each pair of mammalian species (right panel). D Left panels show Hierarchical Clustering Trees of gene expression and Spearman’s correlation coefficients based on 1:1 orthologs across the ten mammal species (7,846 protein coding genes, top panel), 11 amniotes (6,322 genes, middle panel), and 12 vertebrates (4,881 genes, bottom panel) respectively; right panels represent the Pearson’s correlation of similarity in gene expression for each pair of species and their divergence time across ten mammals, 11 amniotes, and 12 vertebrates, respectively.


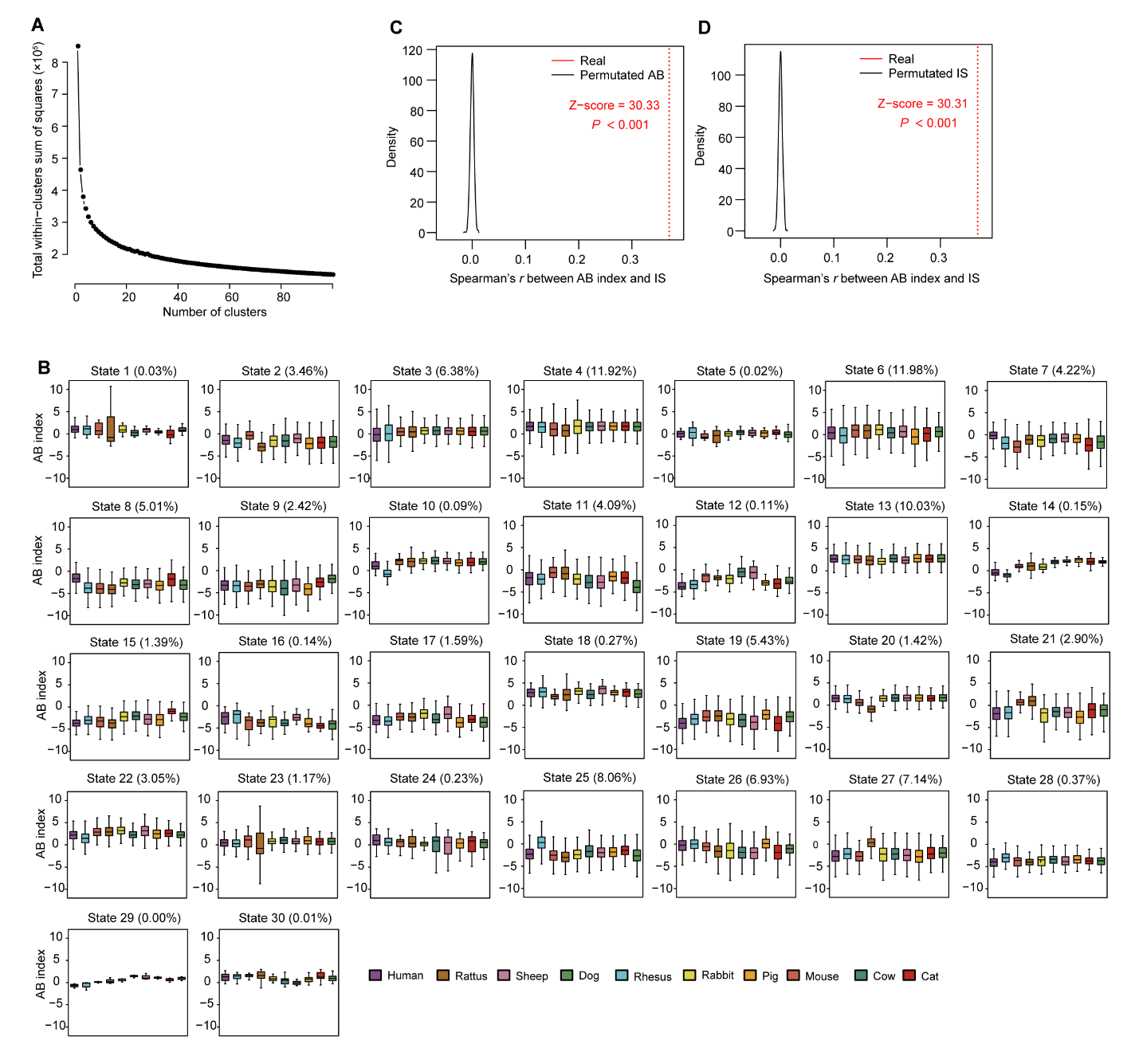


**Fig. S5** Thirty A/B compartment states predicted using Phylo-HMGP. **A** State number estimation from *K*-means clustering. The change of Sum of Squared Error (SSE) with respect to an increased cluster number in *K*-means clustering on AB index data. The state number was estimated between 20 and 40 based on *K*-means clustering results. **B** Different patterns of A/B compartment states across 10 mammalian species predicted by Phylo-HMGP-OU for 30 states. The y-axis represents the AB index value. Box plots of AB index value distributions in each predicted state for the 10 species. The percentage of the number of regions in each predicted state are also shown in the title of each plot. The internal line indicates the median, the box limits indicate the upper and lower quartiles and the whiskers extend to 1.5 IQR from the quartiles. **C, D** The Spearman correlation coefficient between AB index and IS in conserved regions across mammalian species. The real and permutated data are presented as black curves and read dot lines, respectively. The permutation of the AB index and IS are shown in **C** and **D**, respectively. The permutation was performed by 1,000 bootstrap replicates. *P* values were calculated using permutation test.


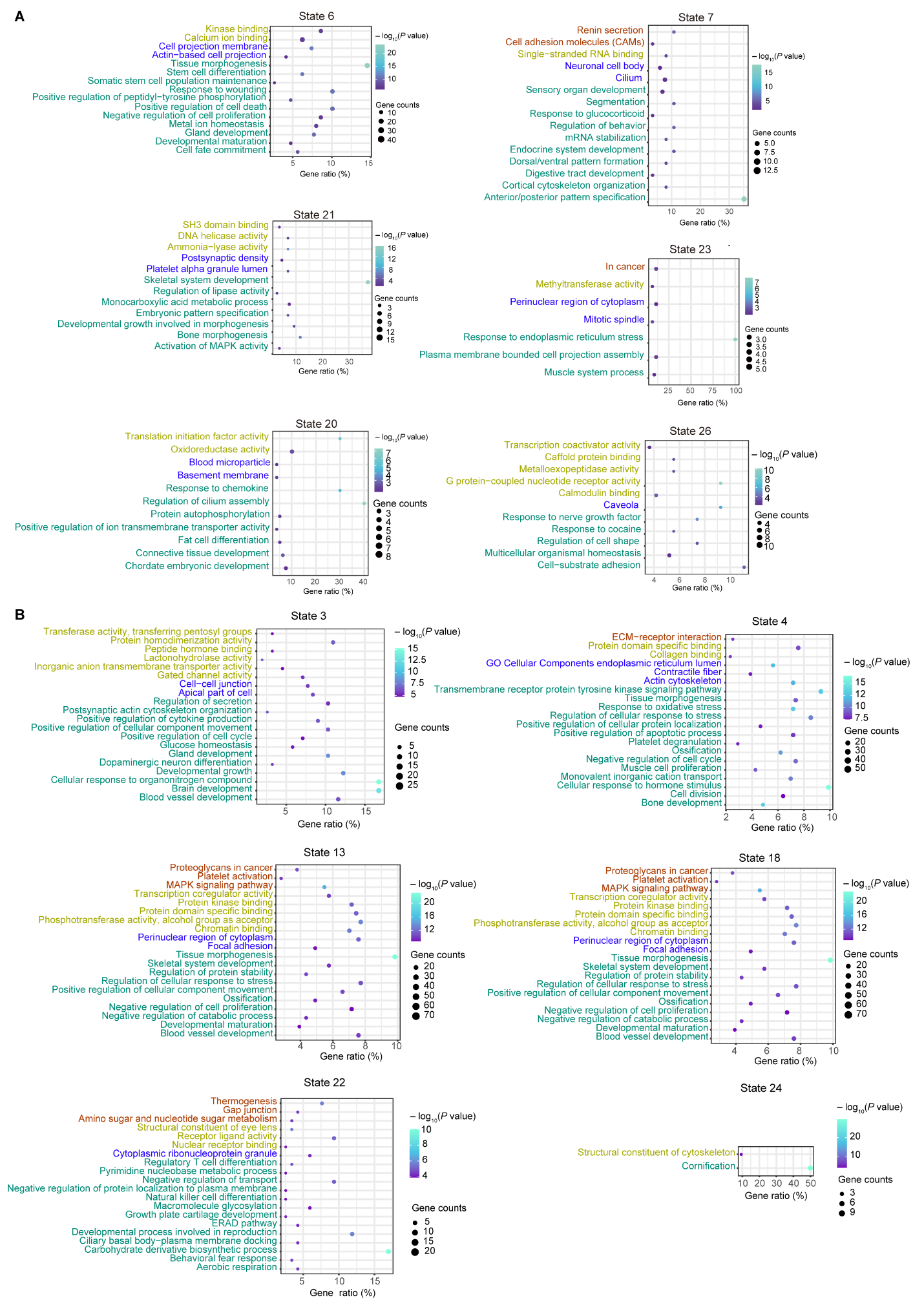


Fig. S6 Gene functions of species-specific compartments and conserved A compartments. Gene ontology (GO) terms or KEGG pathways of states that show signiﬁcant correlation with regions that are Non-conserved A and Conserved A compartments B.


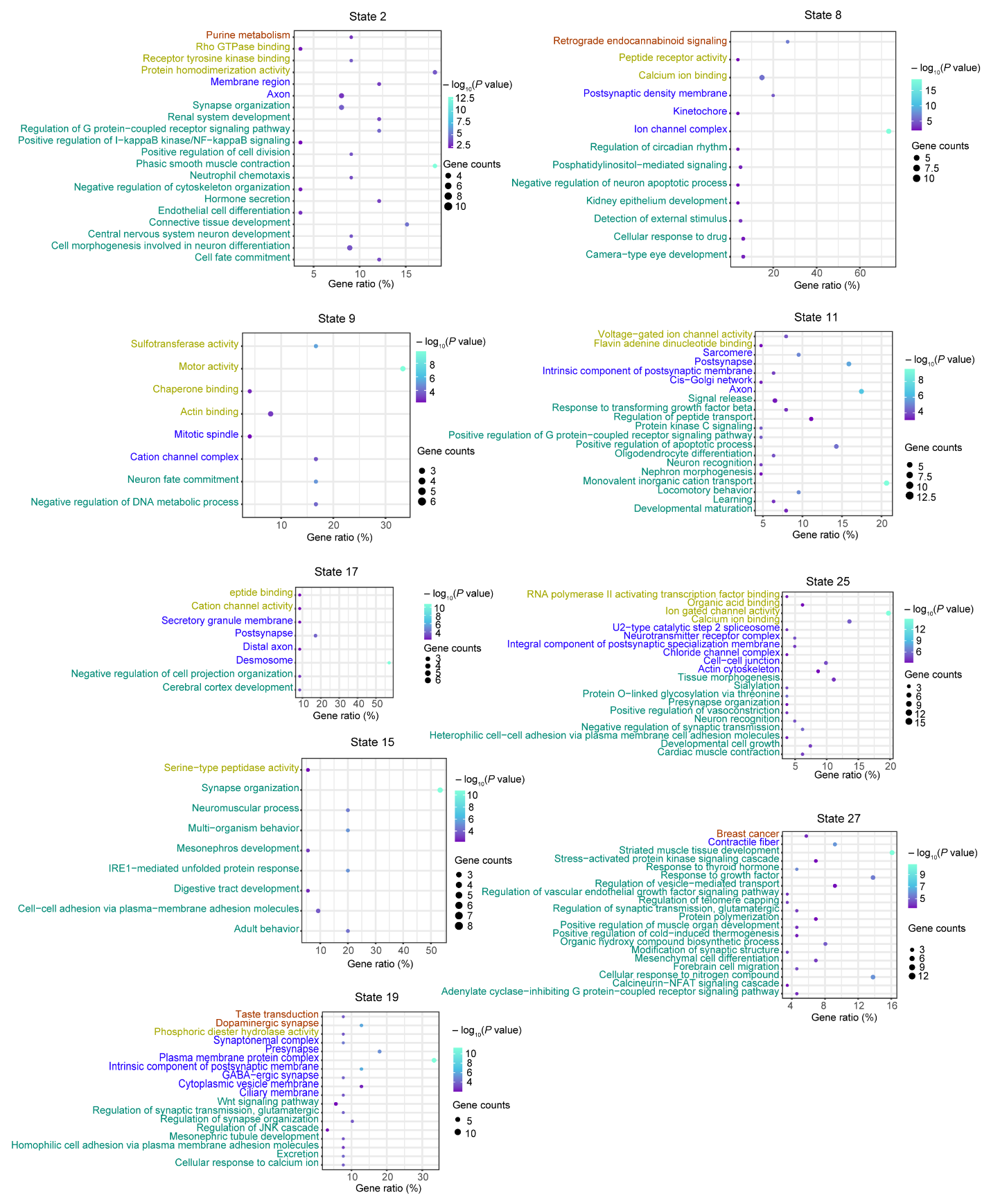


Fig. S7 Gene functions of conserved B compartments. Gene ontology (GO) terms or KEGG pathways of states that showed signiﬁcant correlation with regions that are conserved B compartments.

We performed GO analysis for the 30 compartment states and found clear differences in gene functions of Conserved A/B compartment regions. The Non-conserved Compartments can be identiﬁed as species-speciﬁc states. Those conserved compartment A regions that were mainly involved in development include ‘Gland development’, ‘Development growth’, ‘Brain development’, and ‘Blood vessel development’ for state 3; ‘Bone development’ for state 4; ‘Skeletal system development’ and ‘Blood vessel development’ for state 13; and ‘Growth plate cartilage development’ for state 22 (**Fig. S6B**). Neuron-related categories were mainly enriched in compartment B regions (**Fig. S7**), such as ‘Cell morphogenesis involved in neuron differentiation’ and ‘Central nervous system neuron development’ for state 2; ‘Negative regulation of neuron apoptotic process’ for state 8; ‘Neuron fate commitment’ for state 9; ‘Nephron morphogenesis’ and ‘Neuron recognition’ for state 11; and ‘Neuron recognition’ for state 25.


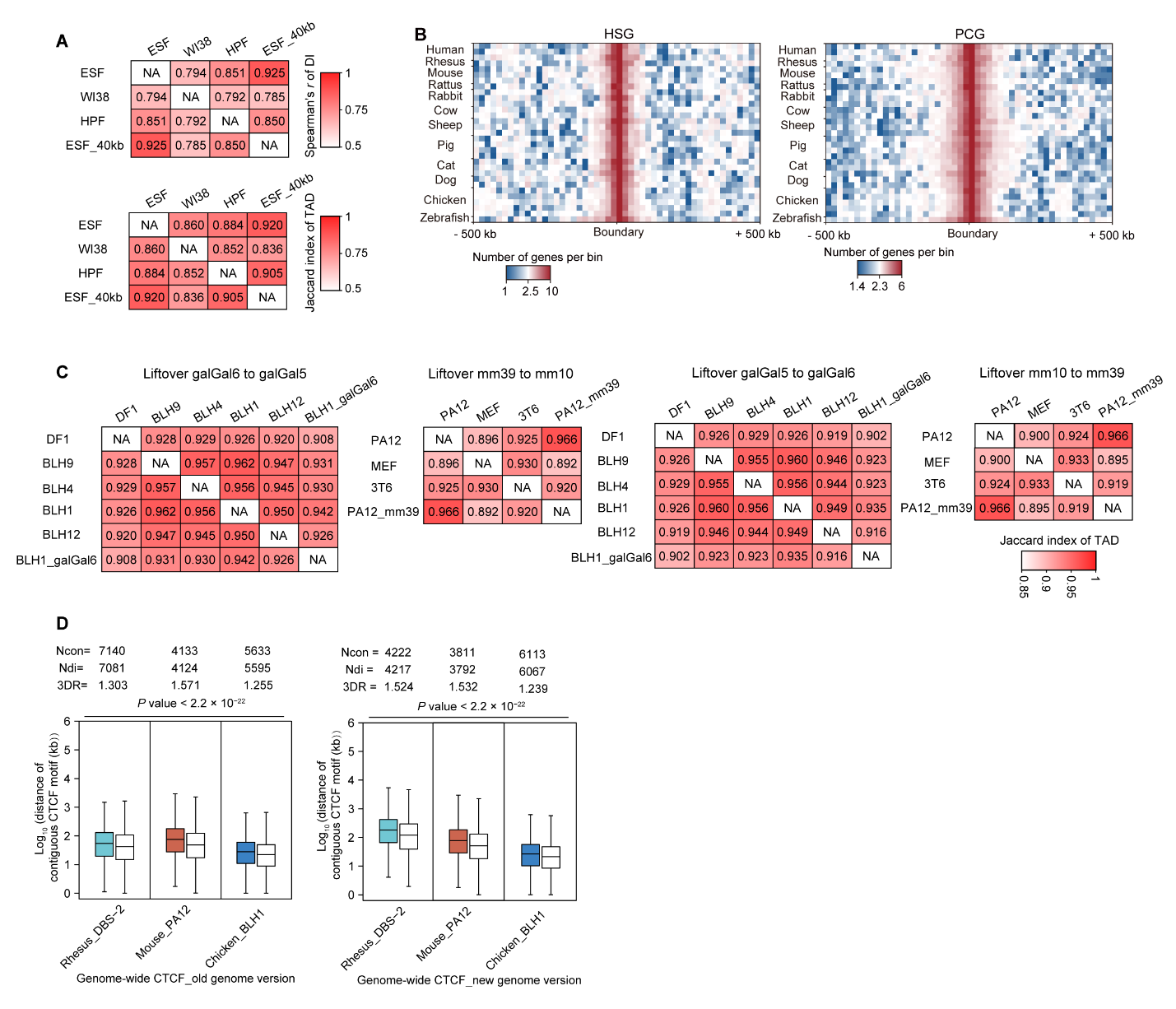


**Fig. S8** TADs are structural and regulatory units conserved across vertebrates. **A** Spearman’s correlation of DI values (top panel) and Jaccard index of TADs (bottom panel) among human samples. ESF_40kb indicates TADs called at 40 kb resolution. The remaining TADs were called at a resolution of 20 kb. **B** Enrichment for housekeeping gene (left panel) and protein coding gene (right panel) TSSs (transcriptional start sites) in TAD boundary regions in 12 species. **C** Similar TAD characteristics between different genome assemblies. Jaccard index of TADs called under old (gal5Gal and mm10) and updated (galGal6 and mm39) genome assembly versions of chicken and mouse. TAD positions at different genome assemblies were liftovered to the same assembly. The jaccard index of rhesus TADs between two genome assemblies were 0.918 (liftover Mmul_8.0.1 TADs to Mmul_10) and 0.920 (liftover Mmul_10 TADs to Mmul_8.0.1), which were not shown as figures. **D** 3DR values of genome under new and old genome assemblies of rhesus, mouse and chicken. The internal line indicates the median, the box limits indicate the upper and lower quartiles and the whiskers extend to 1.5 IQR from the quartiles.


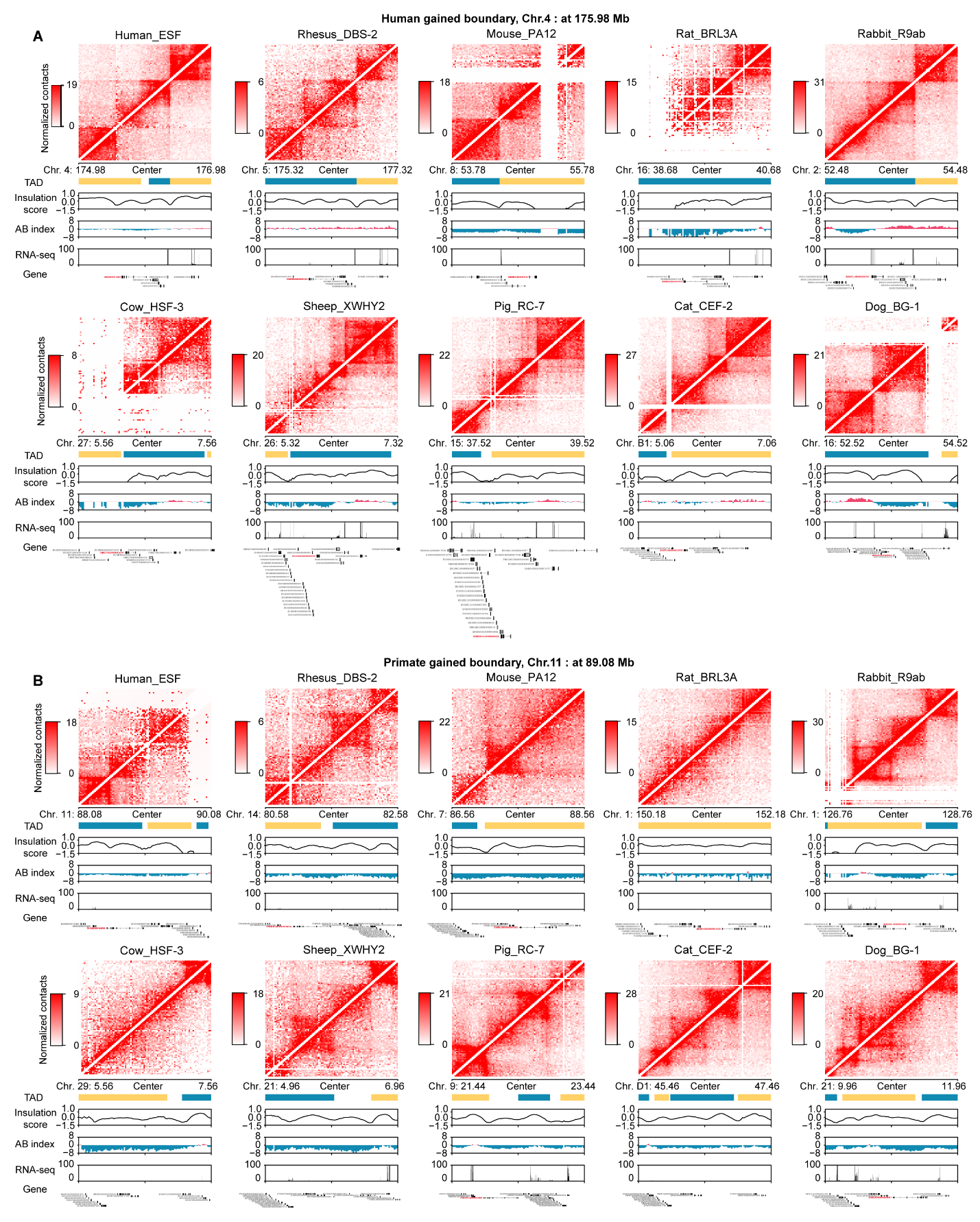


**Fig. S9** Examples of human gained and primate gained TAD boundaries. Hi-C contact maps of human gained **A** and primate gained **B** TAD boundaries correlated with genes *GPM6A* and *GRM5*, respectively. Gene IDs of *GPM6A* and *GRM5* in each species was marked in red.


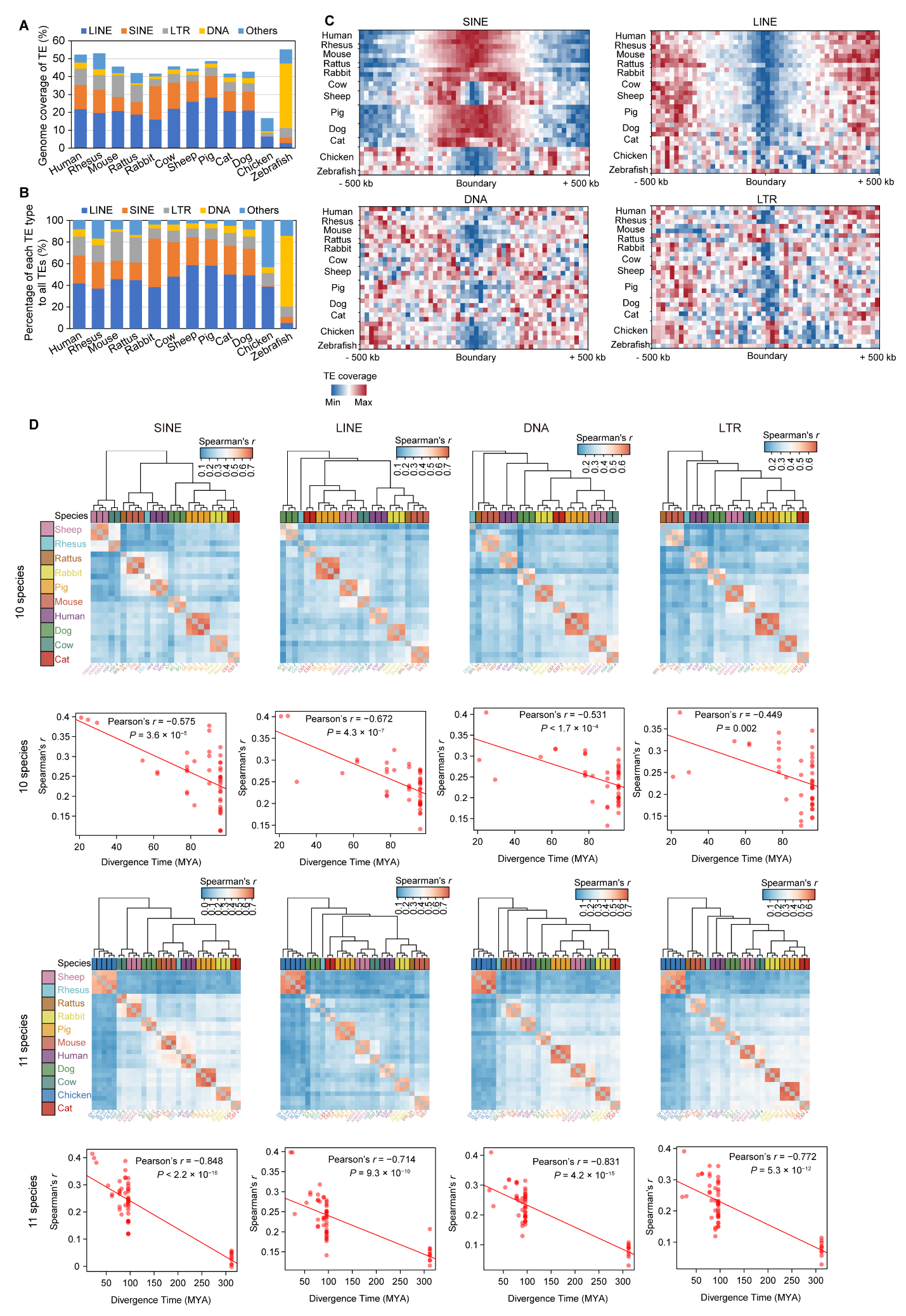


Fig. S10 TEs and genome architecture. A TE content in 12 species. These four TE types typically comprise nearly one half (ranging from ~36.32% for cat to ~47.92% for rhesus) of mammal genomes, whereas only ~9.52% and ~47.36% were identified in chickens and zebrafish. LINE (~39.77% for rabbit to ~59.71% for sheep) and SINE (~18.66% for mouse to ~46.65% for rabbit) retrotransposons are the most common types of TEs in mammals; these LINEs and SINEs take up more than two thirds of mammalian genomes (ranging from 61.2% for rhesus to 84.3% for sheep). However, in zebrafish, DNA transposons make up ~76.27% of the TEs. B Proportions of each TE type among all detected TEs. C Enrichment for 4 TEs in TAD boundary regions in 12 species. D The Spearman correlation of TE coverage across 10 mammals and 11 species; and correlation between similarity of TE coverage in putative enhancer regions and evolutionary divergence time in 10 mammalian (top panels) and 11 amniotes (bottom panels). The similarity of TE coverage in putative enhancer regions were calculated by the Spearman’s *r* between pairwise species.
